# An approach based on linear programming to build experimentally driven pump-leak models

**DOI:** 10.1016/j.bpj.2026.01.021

**Published:** 2026-01-13

**Authors:** Luigi Catacuzzeno, Maurizio G. Cavaliere, Antonio Michelucci

**Affiliations:** 1 Department of Chemistry, Biology, and Biotechnology, University of Perugia, Perugia, Italy

## Abstract

Pump-Leak (P-L) models are powerful tools in membrane and cellular physiology, providing a quantitative framework to understand how cells regulate intracellular ion concentrations, cell volume, and membrane potential thorugh ion transport mechanisms. However, constructing a P-L model for a specific cell type is challenging, because it requires numerous cell-specific parameters, many of which are experimentally inaccessible. Here, we present a linear programming-based method to estimate such parameters, using only a subset of experimentally determined values. This inverse approach exploits the system of differential equations underlying the P-L model and enforces steady-state conditions by setting all time derivatives to zero. Experimentally measured steady-state intracellular ion concentrations, membrane potential, and cell volume are used as constraints, while model parameters are treated as variables. Linear programming is then employed to systematically explore feasible parameter ranges, allowing experimentally determined values to be assigned to selected parameters by progressively restricting the ranges of the remaining ones until convergence to unique solutions is achieved. We applied this strategy to construct a P-L model for the U87-MG glioblastoma cell line. The resulting model accurately predicts several dynamical behaviors, including volume changes induced by reductions in extracellular Na^+^ and by suppression of resting ion conductance. A simple computer application implementing this method is provided, offering a general strategy for parametrizing P-L models in different cell types.

## Significance

Cellular ion homeostasis and volume regulation underpin numerous physiological and pathological processes. Pump-leak (P-L) models provide a powerful quantitative framework for analyzing these mechanisms, but their application has been limited by the difficulty of determining the many cell-specific parameters they require. Here, we present a linear programming-based approach that infers otherwise inaccessible unknown parameters from a limited set of experimentally measured steady-state values. This method enables the construction of data-driven, cell-type-specific P-L models, demonstrated here for U87-MG glioblastoma cells. By providing both a computational implementation and a generalizable parametrization strategy, this work facilitates broader and more rigorous use of P-L models in cellular physiology and biophysical research.

## Introduction

Animal cells are enclosed by a plasma membrane that is permeable to water and small ions but impermeable to many relatively large and negatively charged intracellular solutes, which contribute up to ∼100 mOsm to the cytosolic osmotic balance. The principal impermeant species consist primarily of small organic anions, including ATP and amino acids, rather than macromolecules such as proteins or nucleic acids, and carry an effective mean charge of approximately −1 (1,2). This asymmetry between permeant and impermeant ions establishes a Donnan osmotic imbalance. In the absence of active volume-regulatory mechanisms, the resulting osmotic pressure would drive sustained water influx, leading to progressive cell swelling and ultimately lysis (1,2,3). While plant and bacterial cells counteract the Donnan effect through the structural support of a rigid cell wall, animal cells rely on the pump-leak (P-L) mechanism to maintain volume homeostasis. Tosteson and Hoffman demonstrated that cell volume regulation depends on the energy-dependent extrusion of Na^+^ and uptake of K^+^ via the Na^+^/K^+^-ATPase, balanced by the passive permeability of the plasma membrane to Na^+^, K^+^, and Cl^−^ ions, as well as water (4). Their work established that this coordinated transport system enables cells to maintain stable volume despite the osmotic pressure imposed by intracellular impermeant solutes.

The P-L mechanism establishes a transmembrane asymmetry in Na^+^ and K^+^ concentrations, together with a negative resting membrane potential, typically from −80 to −30 mV. This electrochemical configuration drives Cl^−^ efflux, thereby creating the osmotic space required to accommodate intracellular impermeant anions (5).

This framework is mathematically represented by a system of differential equations that describes the time-dependent evolution of intracellular ion concentrations, membrane potential, and cell volume, incorporating both passive and active transmembrane ion fluxes. The model is constrained by two fundamental biophysical requirements: osmotic equilibrium, which demands equal total solute concentrations across the plasma membrane, and electroneutrality, which enforces near-balance between intracellular positive and negative charges (4,6). Together, these coupled constraints coordinate ion fluxes and water movement, underscoring the central role of the P-L mechanism in maintaining cellular ionic and volume homeostasis.

Given the fundamental physiological importance of cell volume regulation, the P-L model provides critical insights into cellular behavior and the consequences of modulating membrane transport processes. It also offers a foundational framework for understanding how cells maintain homeostasis under both physiological and perturbed conditions. Despite its utility, the application of the P-L model in numerical simulations has remained relatively limited (1,2), largely because accurately determining the full set of required parameters, including the activities and expression levels of membrane transporters and ion channels, poses significant experimental challenges across different cell types.

In contrast, the Hodgkin-Huxley model of membrane excitability (7) has been widely adopted and has supported extensive numerical simulations exploring electrical behavior across diverse cell types (8,9). This broad utilization likely reflects the fact that key Hodgkin-Huxley parameters can be routinely measured using standard electrophysiological techniques. By comparison, constructing a P-L model requires a more comprehensive experimental toolkit spanning ion transport, membrane biophysics, and cell volume regulation capabilities that are not always available within a single laboratory.

In this study, we present a strategy based on linear programming (LP) to address the challenges of constructing a P-L model for specific cell types. Because many model parameters are interdependent, steady-state conditions can, in principle, be used to infer the full set of parameters from a subset that is experimentally accessible. This approach allows unknown parameters to be estimated either precisely or within physiologically plausible ranges, thereby facilitating model construction without requiring exhaustive experimental data. We refer to this strategy as the “LP method” or “inverse method,” as it inverts the conventional modeling paradigm by using known steady-state conditions to infer unknown parameters. To demonstrate its utility, we applied the inverse method to human U87-MG glioblastoma (GBM) cells, a well-established model for studying cell volume regulation (10,11,12). These cells maintain robust ionic and osmotic homeostasis under both physiological and stress conditions, making them particularly suitable for investigating the P-L-mediated mechanisms underlying volume stabilization. To support broader adoption, we provide a user-friendly computational tool as supporting material, enabling implementation in diverse experimental systems.

## Materials and methods

### Theory: P-L model and linear programming

#### The P-L model equations

**Figure 1:**
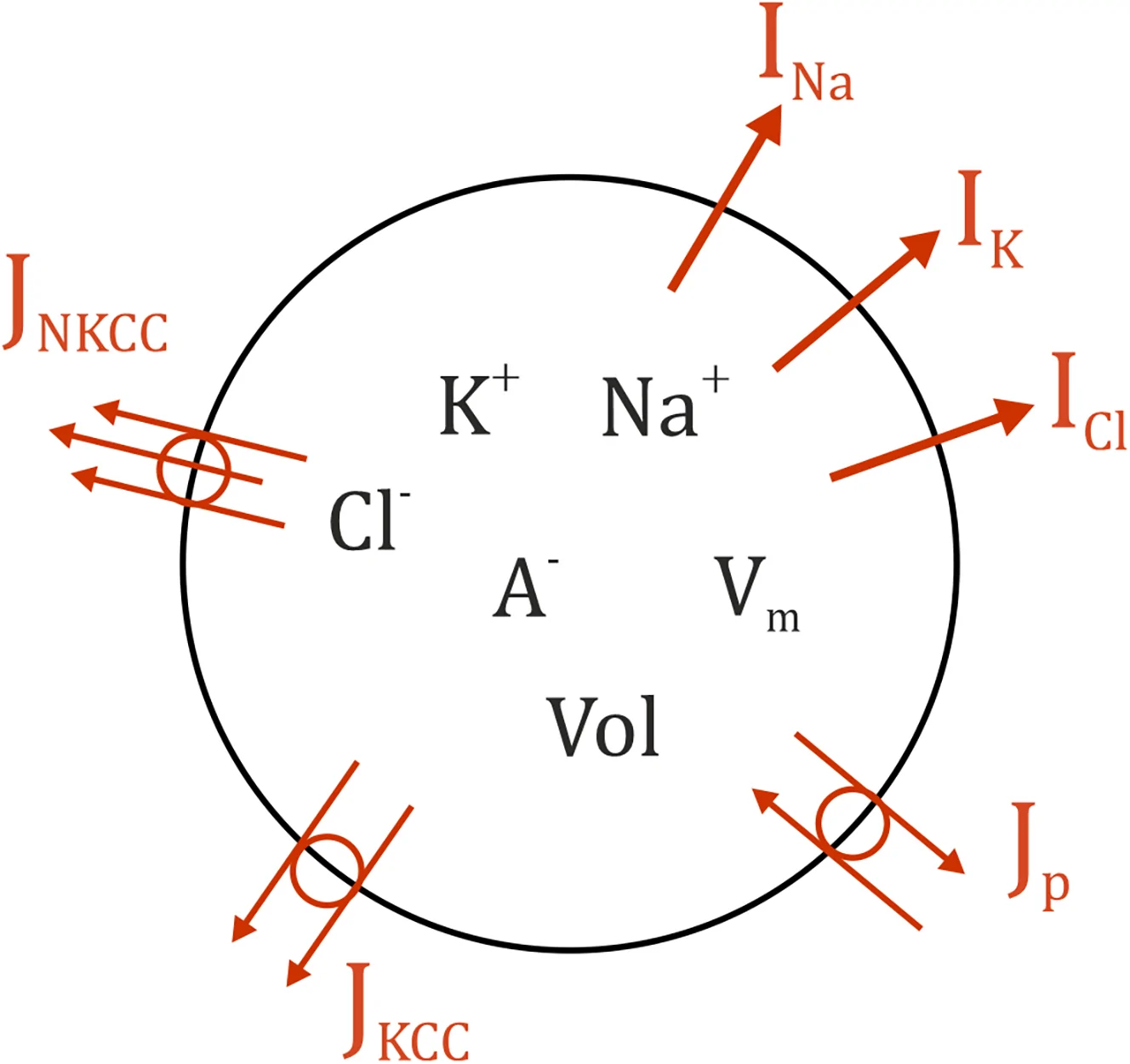
The P-L model. Schematic representation of the main components of the P-L model used in this study. Red arrows indicate ionic transport pathways, with arrow direction corresponding to positive fluxes, including Na^+^ (I_Na_), K^+^ (I_K_), and Cl^−^ (I_Cl_) conductances, the Na^+^/K^+^^-^ATPase (J_p_); the K^+^/Cl^−^ cotransporter (KCC; J_KCC_); and the Na^+^/K^+^/2Cl^−^ cotransporter (NKCC; J_NKCC_). The circle represents the cell plasma membrane. Model variables, shown in black, include intracellular concentrations of Na^+^, K^+^, and Cl^−^, the concentration of impermeable anions (*A*^−^), the membrane potential (*V*_*m*_), and the cell volume (*Vol*).

The present model extends the classical framework introduced by Tosteson and Hoffman (4) by incorporating additional ion transport pathways, as described by Aminzare and Kay (13). We consider a simplified cellular system enclosed by an extensible plasma membrane, permeable to Na^+^, K^+^, Cl^−^, and water, and bathed in an extracellular solution of constant ionic composition (Fig. 1).

Intracellular ionic concentrations and membrane potential (*V*_*m*_) are assumed to be spatially uniform, with no cytoplasmic gradients. Ion transport across the membrane occurs either passively, through ion channels following their electrochemical gradients, or actively, via energy-dependent transporters. Specifically, the Na^+^/K^+^-ATPase is modeled as the sole primary active transporter, exporting three Na^+^ ions and importing two K^+^ ions per cycle. Passive fluxes are mediated by Na^+^, K^+^, and Cl^−^ channels, whereas electroneutral cotransport is facilitated by the Na^+^/K^+^/2Cl^−^ cotransporter (NKCC) and the K^+^/Cl^−^ cotransporter (KCC). Collectively, these transport mechanisms regulate the intracellular number of moles (*n*) of permeant ions (*n*_*Na*_, *n*_*K*_, *n*_*Cl*_) and influence the *V*_*m*_. Osmotically driven water fluxes, arising from intracellular-extracellular osmotic imbalances, produce changes in cell volume (*Vol*). The temporal evolution of these five state variables (*n*_*Na*_, *n*_*K*_, *n*_*Cl*_, *V*_*m*_, *Vol*) is governed by the following set of P-L differential equations:

(1) $$\frac{dn_{Na}}{dt}=-\frac{I_{Na}}{F}-3\;J_{p}-J_{NaK2Cl}$$

(2) $$\frac{dn_{K}}{dt}=-\frac{I_{K}}{F}+2\;J_{p}-J_{NaK2Cl}-J_{KCl}$$

(3) $$\frac{dn_{Cl}}{dt}=\frac{I_{Cl}}{F}-2\;J_{NaK2Cl}-J_{KCl}$$

(4) $$\frac{dV_{m}}{dt}=-\frac{I_{Na}+I_{K}+I_{Cl}+F\;J_{p}}{C_{m}}$$

(5) $$\frac{dVol}{dt}=P_{w}\;\left(\Pi_{i}-\Pi_{ex}\right)$$

The first three equations (Eqs. 1, 2, and 3) describe time-dependent changes in intracellular ion content, governed by the combined activity of membrane transporters. *C*_*m*_ and *F* denote the membrane electrical capacitance and Faraday’s constant, respectively. The terms *I*_*Na*_, *I*_*K*_, and *I*_*Cl*_ correspond to the passive Na^+^, K^+^, and Cl^−^ membrane currents under resting conditions, respectively. They are modeled as follows:

$$I_{Na}=g_{Na}\;{Na}_{in}\left(V-E_{Na}\right),\;with\;E_{Na}=\frac{RT}{zF}\ln\frac{{Na}_{ex}}{{Na}_{in}}$$

$$I_{K}=g_{K}\;K_{in}\;\left(V-E_{K}\right),\;with\;E_{K}=\frac{RT}{zF}\ln\frac{K_{ex}}{K_{in}}$$

$$I_{Cl}=g_{Cl}\;{Cl}_{in}\;\left(V-E_{Cl}\right),\;with\;E_{Cl}=\frac{RT}{zF}\ln\frac{{Cl}_{in}}{{Cl}_{ex}}$$

Here, *g*_*Na*_, *g*_*K*_, and *g*_*Cl*_ are the resting conductances for Na^+^, K^+^, and Cl^−^, respectively. The intracellular concentrations of Na^+^, K^+^, and Cl^−^ are defined as ${Na}_{in}=\frac{n_{Na}}{Vol}$, $K_{in}=\frac{n_{K}}{Vol}$, and ${Cl}_{in}=\frac{n_{Cl}}{Vol}$, whereas *Na*_*ex*_, *K*_*ex*_, and *Cl*_*ex*_ denote the corresponding extracellular concentrations. These linear expressions for passive ion currents represent approximations of the Goldman-Hodgkin-Katz flux equation, valid under the assumption of small, near-resting membrane potentials and constant extracellular ion concentrations during the computation (14). In this formulation, conductance parameters are normalized to the intracellular concentration of the corresponding permeant ion, allowing ionic currents to adjust appropriately as intracellular concentrations change over time. Importantly, conductances are not normalized to membrane surface area, unlike the convention commonly adopted in electrophysiological models (15). Because the model explicitly incorporates cell volume changes driven by water flux, and because the plasma membrane of a typical animal cell contains numerous folds and invaginations, volume increases primarily occur through membrane unfolding rather than changes in total surface area. Consequently, the effective membrane area available for ion channels does not scale with the spherical surface used to approximate cell geometry. Normalizing conductances to this geometric surface would introduce an artificial and physiologically unrealistic dependence of conductance on cell volume, implying that swollen cells have higher conductance simply due to increased surface. To avoid this “artifact”, membrane ionic conductances in our model are expressed independently of membrane surface area.

The term *J*_*p*_ represents the Na^+^/K^+^ ATPase flux, which is modeled as

$$J_{p}=\frac{Q_{p}}{1.0+{\left(\frac{k_{p}}{{Na}_{in}}\right)}^{3}}\text{,}$$

where *Q*_*p*_ is the maximal Na^+^/K^+^ ATPase flux, and *k*_*p*_ denotes the apparent affinity of the pump for intracellular Na^+^. This formulation omits the extracellular K^+^ binding term, which is assumed to remain constant under our model assumptions and is therefore incorporated into *Q*_*p*_. The NKCC flux is represented by

$$J_{NKCC}=Q_{NKCC}\left({Na}_{in}\;K_{in}\;{Cl}_{in}^{2}-{Na}_{ex}\;K_{ex}\;{Cl}_{ex}^{2}\right)\text{,}$$

where *Q*_*NKCC*_ is the flux at unitary ion concentration differences. Similarly, the KCC flux is described by

$$J_{KCC}=Q_{KCC}\left(K_{in}\;{Cl}_{in}-K_{ex}\;{Cl}_{ex}\;\right)\text{,}$$

where *Q*_*KCC*_ is the transporter flux at unitary ion concentration differences.

Eq. 4 describes the dynamics of *V*_*m*_ resulting from electrogenic fluxes through Na^+^, K^+^, and Cl^−^ leak channels, together with the Na^+^/K^+^-ATPase, by assimilating the membrane to an RC circuit and applying Kirchhoff’s current law to account for the balance of transmembrane ionic currents. Since changes in membrane potential occur on a much faster timescale than changes in intracellular ion content and volume, we assume a quasi-steady state for *V*_*m*_ in all simulations, following the approach reported in (16). Under this assumption, the equilibrium *V*_*m*_ is given by

$$V_{m}=\frac{g_{Na}\;{Na}_{in}\;E_{Na}+g_{K}\;K_{in}E_{K}+g_{Cl}\;{Cl}_{in}\;E_{Cl}-J_{p}\;F\;}{g_{Na}\;{Na}_{in}+g_{K}\;K_{in}+g_{Cl}\;{Cl}_{in}}$$

Alternatively, the algebraic formulation *V*_*m*_ = *Q*/*C*, which directly relates total charge to the membrane potential, could be used as in (17).

Finally, Eq. 5 describes osmotic water flux across the plasma membrane, driven by the difference between intracellular and extracellular osmolarity. *P*_*w*_ is the plasma membrane water permeability coefficient, Π_*i*_ is the intracellular osmolarity, and Π_*ex*_ is the extracellular osmolarity, respectively, defined as

$$\Pi_{i}={\text{Na}}_{in}+K_{in}+{Cl}_{in}+A^{-}\text{,}$$

where $A^{-}=\frac{n_{A^{-}}}{Vol}$ is the concentration of impermeable cytosolic anions, and

$$\Pi_{ex}={\text{Na}}_{ex}+K_{ex}+{Cl}_{ex}$$

Regarding the impermeant intracellular negative solutes *A*^−^, the model treats their total moles, rather than their concentrations, as the invariant quantity. Consequently, as cell volume (*Vol*) changes, the intracellular *A*^−^concentration is updated automatically.

### The P-L model at steady-state

A particularly important condition of the P-L model is the steady state, which represents the physiological equilibrium achieved by a cell after a prolonged unperturbed period. This resting state is of central interest, as it provides a well-defined physiological baseline. Intracellular ion concentrations, *V*_*m*_, and cell volume at steady state are typically available in the literature or can be measured experimentally. Table 1 summarizes the steady-state variables used in our model for GBM cells, based on previously reported data or values obtained in our experiments (see below).

**Table 1:** P-L variable values at steady state for GBM cells and extracellular ion conditions used in our experiments and simulations

Variable	Value	Note
${Na}_{in}^{ss}$	0.01 M	Rose and Ramson (18); GBM cells have Na_i_ similar to astrocytes: Khan et al. (19)
$K_{in}^{ss}$	0.14 M	chosen to set the intracellular osmolarity equal to the extracellular osmolarity
${Cl}_{in}^{ss}$	0.1 M	Habela et al. (20)
$A_{in}^{ss}$	0.05 M	chosen to give electroneutrality
*V*^*ss*^	−0.04 V	experimentally determined; see Fig. 2, *A* and *B*
*Vol*^*ss*^	4.18 × 10^−15^ m^3^	experimentally determined; see Fig. 2*D*
Extracellular condition in which our volume perturbation experiments are performed		
*Na*_*ex*_	0.145 M	
*K*_*ex*_	0.005 M	
*Cl*_*ex*_	0.15 M	

Assuming that the P-L model accurately represents the behavior of the cell type under study, a resting, unperturbed cell maintains stable intracellular ion concentrations, *V*_*m*_, and volume. Under these conditions, all time derivatives in Eqs. 1, 2, 3, 4, and 5 are zero, reducing the system to five algebraic equations. This steady-state system can be solved to determine the corresponding variables, hereafter denoted with the subscript “*ss*.”

(6) $$-\frac{I_{Na}}{F}-3\;J_{p}-J_{NaK2Cl}=0$$

(7) $$-\frac{I_{K}}{F}+2\;J_{p}-J_{NaK2Cl}-J_{KCl}=0$$

(8) $$\frac{I_{Cl}}{F}-2\;J_{NaK2Cl}-J_{KCl}=0$$

(9) $$I_{Na}+I_{K}+I_{Cl}+F\;J_{p}=0$$

(10) $$P_{w}\;\left(\Pi_{i}-\Pi_{ex}\right)=0$$

with

$$I_{Na}=g_{Na}\;{Na}_{in}^{ss}\left(V^{ss}-E_{Na}\right),\;with\;E_{Na}=\frac{RT}{F}\ln\left(\frac{{Na}_{ex}}{{Na}_{in}^{ss}}\right)\text{,}$$

$$I_{K}=g_{K}\;K_{in}^{ss}\;\left(V^{ss}-E_{K}\right),\;with\;E_{K}=\frac{RT}{F}\ln\left(\frac{K_{ex}}{K_{in}^{ss}}\right)\text{,}$$

$$I_{Cl}=g_{Cl}\;{Cl}_{in}^{ss}\;\left(V^{ss}-E_{Cl}\right),\;with\;E_{Cl}=\frac{RT}{F}\ln\left(\frac{{Cl}_{in}^{ss}}{{Cl}_{ex}}\right)$$

$$J_{p}=\frac{Q_{p}}{1.0+{\left(\frac{k_{p}}{{Na}_{in}^{ss}}\right)}^{3}},$$

$$J_{NaK2Cl}=Q_{NaK2Cl}\left({Na}_{in}^{ss}\;K_{in}^{ss}\;{{Cl}_{in}^{ss}}^{2}-{Na}_{ex}\;K_{ex}\;{Cl}_{ex}^{2}\right)\text{,}$$

$$J_{KCl}=Q_{KCl}\left(K_{in}^{ss}\;{Cl}_{in}^{ss}-K_{ex}\;{Cl}_{ex}\;\right),$$

$$\Pi_{i}={Na}_{in}^{ss}+K_{in}^{ss}+{Cl}_{in}^{ss}+A_{in}^{ss},\Pi_{ex}={\text{Na}}_{ex}+K_{ex}+{Cl}_{ex}$$

Directly solving the system of Eqs. 6, 7, 8, 9, and 10 is challenging due to the large number of parameters required by the P-L model, as summarized in Table 2. However, because experimental measurements of the steady-state variables are available or easily obtainable, we propose an alternative approach. Rather than solving for the steady-state variables themselves, these variables are treated as known constants and a selected subset of unknown model parameters is inferred from the system of Eqs. 6, 7, 8, 9, and 10.

**Table 2:** Parameters of the P-L model, and their values in U87-MG cells, determined as described in the results

Parameter	Description	Value in U87-MG cells
*g*_*Na*_	Na^+^ channel conductance	17.78 nS/M
*g*_*K*_	K^+^ channel conductance	3.752 nS/M
*g*_*Cl*_	Cl^−^ channel conductance	4.523 nS/M
*Q*_*p*_	maximal rate of the Na^+^/K^+^ ATPase	1.77 × 10^−4^ pmol/s
*Q*_*NKCC*_	rate of the NKCC at unitary ion concentrations	31.6 pmol/(s M^4^)
*Q*_*KCl*_	rate of the KCC at unitary ion concentrations	5.54 × 10^−4^ pmol/(s M^2^)
*P*_*w*_	Plasma membrane water permeability	2.14 × 10^−16^ m^3^/s^∗^M
*kp*	Na^+^ binding constant for the Na/K pump	9.8 mM

Notably, the first seven parameters listed in Table 2 depend on the abundances of specific ion transporters and are therefore highly cell-type specific. By treating these seven parameters as unknown variables, the steady-state system (Eqs. 6, 7, 8, 9, and 10) is reduced to a linear system. Specifically, an equation of the form *f*(*x*_1_,*x*_2_,*x*_3_, … …) = 0 is considered linear in its variables *x*_1_,*x*_2_,*x*_3_, … … if it can be expressed as ${a_{0}+a}_{1}\;x_{1}+a_{2}\;x_{2}+a_{3}\;x_{3}\ldots \ldots ..=0$, where *a*_0_, *a*_1_,*a*_2_,*a*_3_ … are known constants.

Linearity does not hold for the parameter *kp*, which represents the intracellular Na^+^ binding affinity of the Na^+^/K^+^-ATPase. However, *kp* is typically well characterized in the literature, as its value tends to be conserved across cell types expressing the same isoform of the transporter. Alternatively, it can be measured experimentally if greater precision is required.

Finally, because a resting cell is at osmotic equilibrium (i.e., Π_*i*_ = Π_*ex*_), Eq. 10 is undetermined, meaning that any value of *P*_*w*_ satisfies the equation. Thus, *P*_*w*_ does not influence the steady-state condition but only governs the kinetics of volume equilibration. Therefore, *P*_*w*_ cannot be estimated from steady-state data alone. Taking these considerations into account, the system reduces to four equations containing six linear variables/unknowns, which are highlighted in bold below:

(11) $$-\frac{g_{Na}\;{Na}_{in}^{ss}\left(V^{ss}-E_{Na}\right)}{F}-\frac{3Q_{p}}{1.0+{\left(\frac{k_{p}}{{Na}_{in}^{ss}}\right)}^{3}}-Q_{NaK2Cl}\left({Na}_{in}^{ss}\;K_{in}^{ss}\;{{Cl}_{in}^{ss}}^{2}-{Na}_{ex}\;K_{ex}\;{Cl}_{ex}^{2}\right)=0$$

(12) $$-\frac{g_{K}\;K_{in}^{ss}\;\left(V^{ss}-E_{K}\right)}{F}+\frac{{2\;Q}_{p}}{1.0+{\left(\frac{k_{p}}{{Na}_{in}^{ss}}\right)}^{3}}-Q_{NaK2Cl}\left({Na}_{in}^{ss}\;K_{in}^{ss}\;{{Cl}_{in}^{ss}}^{2}-{Na}_{ex}\;K_{ex}\;{Cl}_{ex}^{2}\right)-Q_{KCl}\left(K_{in}^{ss}\;{Cl}_{in}^{ss}-K_{ex}\;{Cl}_{ex}\;\right)=0$$

(13) $$\frac{g_{Cl}\;{Cl}_{in}^{ss}\;\left(V^{ss}-E_{Cl}\right)}{F}-2\;Q_{NaK2Cl}\left({Na}_{in}^{ss}\;K_{in}^{ss}\;{{Cl}_{in}^{ss}}^{2}-{Na}_{ex}\;K_{ex}\;{Cl}_{ex}^{2}\right)-Q_{KCl}\left(K_{in}^{ss}\;{Cl}_{in}^{ss}-K_{ex}\;{Cl}_{ex}\;\right)=0$$

(14) $$g_{Na}\;{Na}_{in}^{ss}\left(V^{ss}-E_{Na}\right)+g_{K}\;K_{in}^{ss}\;\left(V^{ss}-E_{K}\right)+g_{Cl}\;{Cl}_{in}^{ss}\;\left(V^{ss}-E_{Cl}\right)+F\;\frac{Q_{p}}{1.0+{\left(\frac{k_{p}}{{Na}_{in}^{ss}}\right)}^{3}}=0$$

Although a linear system of four equations with six unknowns is inherently underdetermined and admits infinitely many solutions, analysis using LP techniques can provide valuable insights into the feasible value ranges of the parameters. Moreover, the LP approach allows assessment of whether the addition of experimental constraints renders the system fully determined, as demonstrated below.

### Linear programming

LP is a mathematical optimization technique used to determine the optimal outcome of a system subject to a defined set of linear constraints. LP has broad applications across diverse fields, including operations research (i.e., resource allocation, scheduling, and production planning), economics (i.e., profit maximization and cost minimization), engineering (i.e., network design and flow optimization), and finance (e.g., portfolio optimization and investment strategies). Although less commonly applied in biology, LP has also been successfully used in several biological contexts (21,22). In its general form, LP determines the values of *N* independent variables (*x*_1_,*x*_2_, … … …,*x*_*N*_) that *maximize* a linear objective function:

$${z=a_{01}\;x}_{1}+a_{02}{\;x}_{2}+\ldots \ldots \ldots +a_{0N}\;x_{N}$$

subject to *M* = *m*_1_ + *m*_2_ + *m*_3_ linear constraints:

$${a_{i1}\;x}_{1}+a_{i2}{\;x}_{2}+\ldots +a_{iN}\;x_{N}\leq b_{i},\;with\;i=1,2,\ldots .,m_{1}\text{,}$$

$${a_{j1}\;x}_{1}+a_{j2}{\;x}_{2}+\ldots +a_{jN}\;x_{N}\geq b_{j},\;with\;j=m_{1}+1,m_{1}+2,\ldots .,{m_{1}+m}_{2}\text{,}$$

$${a_{k1}\;x}_{1}+a_{k2}{\;x}_{2}+\ldots +a_{kN}\;x_{N}=b_{k},\;with\;j={m_{1}+m}_{2}+1,\ldots .,{m_{1}+m}_{2}+m_{3}\text{.}$$

The coefficients *a*_*ij*_ and constants *b*_*i*_ may be positive, negative, or zero. The relative numbers of constraints (*M*) and unknowns (*N*) do not inherently affect the solvability of the LP problem. The linear function *z* to be maximized is called the “objective function.” A solution (i.e., a set of $x_{i}^{\prime }$ that maximizes *z*) may fail to exist if the constraints are mutually incompatible or if the objective function is unbounded, meaning that one or more variables can increase indefinitely while still satisfying all constraints. LP problems can be efficiently solved using the Simplex method, first proposed by Dantzig in 1947 (23). Numerous programming libraries and standalone software packages, including free options, implement this algorithm and solve LP problems. In this study, we developed a custom C implementation of the Simplex algorithm, based on the LP algorithm described by Press and colleagues (24), optimized specifically for parameter analysis of the P-L model. The source code is provided as supporting material.

In the context of the P-L model, the LP approach was applied to determine the permissible ranges of six linear parameters (*g*_Na_, *g*_K_, *g*_Cl_, *Q*_*p*_, *Q*_NKCC_, *Q*_KCC_), under steady-state conditions. The steady-state Eqs. 6, 7, 8, and 9, which enforce charge conservation and osmotic equilibrium, were expressed as equality constraints. For each parameter *x*_*i*_, two LP problems were solved to maximize and minimize their value as follows:

$${z_{i,\max}=x}_{i}\;\text{or}\;{z_{i,\min}=-x}_{i}\;i=1,\ldots .,N\text{.}$$

$$z_{i,\min}\leq x_{i}\leq z_{i,\max}$$

This procedure yields the feasible range consistent with ionic equilibrium, osmotic balance, and electroneutrality.

To address the underdetermined nature of the system, we implemented an iterative LP procedure in which experimentally measured or literature-derived parameters were sequentially introduced as equality constraints. When all six parameters were initially free, the feasible solution space was unbounded, reflecting that the steady-state equations alone do not uniquely determine conductances and fluxes. Introducing a single experimentally determined parameter (i.e., *g*_*Na*_) imposed an additional constraint, and the LP problem was re-solved for the remaining unknowns to identify updated feasible ranges. Each iteration progressively reduced the dimensionality of the feasible region until the solution space collapsed to a single point, defining a unique parameter set consistent with both the steady-state model and experimental data.

The complete sequence of LP-derived parameter bounds is summarized in Table 4 and detailed in the section application of the LP method to determine the full set of P-L model parameters. All calculations can be reproduced using the accompanying C code and the compiled Windows executable provided in the supporting material. Briefly, the LP procedure followed the Simplex algorithm of Press et al. (1992) (24). Once the full parameter set was determined, the system of differential equations representing the P-L model was solved using a Runge-Kutta algorithm.

### Experimental methods

#### Patch-clamp electrophysiology

Whole-cell patch-clamp recordings were performed using both the dialyzed and perforated configuration. Currents were amplified with a HEKA EPC-10 amplifier (HEKA Elektronik), digitized via a 12-bit A/D converter (TL-1 DMA interface, Axon Instruments), and analyzed using PatchMaster software (v2.60, HEKA Elektronik) and Origin 8.0 (OriginLab). Macroscopic currents were low-pass filtered at 3 kHz and sampled at 20 kHz (50 μs per point) during acquisition. Patch pipettes (3–5 MΩ) were pulled from borosilicate glass capillaries (OD 1.5 mm, ID 0.84 mm; World Precision Instruments) using a PUL-100 micropipette puller (World Precision Instruments). The dialyzed whole-cell configuration was used to assess leak conductances and resting *V*_*m*_. Access resistances ranged from 5 to 15 MΩ and electrical access was achieved by mechanical disruption of the patched membrane. Internal solution contained (in mM): KCl 155, EGTA-K 1, MgCl_2_ 1, and HEPES 5 (pH 7.2). The perforated configuration was used to measure resting IK, BK, and VRAC conductances, as well as Na^+^/K^+^-ATPase activity. Access resistances ranged from 15 to 30 MΩ and were achieved within 10–15 min after seal formation by including amphotericin B (200 μM) in the pipette. Internal solution contained (in mM): K_2_SO_4_ 57.5, KCl 55, MgCl_2_ 5, and HEPES 10 (pH 7.2). For both configurations, the extracellular Ringer’s solution contained the following (in mM): 140 NaCl, 5 KCl, 2 CaCl_2_, 2 MgCl_2_, 5 HEPES, and 10 glucose, pH adjusted to 7.4 with NaOH; when leak conductances were assessed, Na^+^ was completely replaced by the impermeant cation choline^+^ to preserve electroneutrality. Octanol (1 mM) was included in all recordings to block gap junctions, electrically isolating the patched cell from neighboring cells. Experiments were conducted at room temperature (18°C–22°C). Conductances were measured in nS and normalized to the total cell membrane capacitance (*C*_*m*_, pF), yielding values in nS/pF. Normalization to capacitance provides an electrical measure of the cell surface and is conceptually equivalent to expressing conductances per unit membrane area (S/cm^2^). For clarity, values expressed as nS/pF can be converted to S/cm^2^, assuming a fixed membrane area, using the relation 1 nS/pF ≈ 10^−3^ S/cm^2^, as reported in Table 3.

**Table 3:** Electrophysiological properties of U87-MG cells

Conductance	nS/pF (or 10^−3^ S/cm^2^) ± SD; sample size (*n*)	*C*_*m*_ (pF) ± SD	[ion]_int_ (M)	nS/M
$g_{Na}^{L}$	0.00504 ± 0.0032; *n* = 8	35.3 ± 10.7	0.01	17.78
$g_{K}^{L}$	0.00432 ± 0.0025; *n* = 8	35.3 ± 10.7	0.145	1.052
$g_{Cl}^{L}$	0.0044 ± 0.0039; *n* = 8	35.3 ± 10.7	0.158	0.983
$g_{K}^{BK}$	0.0064 ± 0.0012; *n* = 6	36.3 ± 7.0	0.17	1.36
$g_{K}^{IK}$	0.0062 ± 0.0027; *n* = 5	36.8 ± 3.7	0.17	1.34
$g_{Cl}^{VRAC}$	0.146 ± 0.0035; *n* = 5	38.4 ± 4.1	0.158	3.54
${g_{Na}=g}_{Na}^{L}$				17.78
$g_{K}=g_{K}^{L}+g_{K}^{BK}+g_{K}^{IK}$				3.75
$g_{Cl}=g_{Cl}^{L}+g_{Cl}^{VRAC}$				4.52

Leak Na^+^, K^+^, and Cl⁻ conductances ($g_{Na}^{L},g_{K}^{L}\text{,}$ and $g_{Cl}^{L}$), as well as basal BK, IK, and VRAC conductances ($g_{K}^{BK}$, $g_{K}^{IK}$, and $g_{Cl}^{VRAC}$) under resting conditions, as estimated from the experiments shown in Figs. 3 and 4. Conductances are expressed in nanosiemens (nS) and reported as conductance densities (nS/pF), normalized to the total cell membrane capacitance (C_*m*_) expressed in picofarad (pF), Given that 1 nS/pF ≈ 10^-3^S/cm^2^, this unit is indicated in parentheses alongside the nS/pF values, assuming a fixed membrane area. Intracellular ion concentrations [ion]_int_ are expressed in molar (M).

#### Cell volume measurements

U87-MG cells were enzymatically detached with trypsin and replated 10–15 min before experiments to induce a transient rounded morphology. Near-spherical cells were selected for analysis enabling accurate volume estimation from two-dimensional projected areas using a spherical approximation. Only cells with projected areas and radii within ±10% of the population mean were included.

Cell volume changes were monitored using video imaging during continuous superfusion with isotonic Ringer’s solution (composition identical to that used for patch-clamp recordings, excluding octanol), as previously described (10,11,12,25). Images were acquired every 20 s using a 40× objective on an upright microscope equipped with a Zeiss Axiocam Cm1 camera and stored in TIFF format. Projected cell area was measured using ImageJ (NIH), and relative cell area (*A*_*rel*_) was calculated as the ratio of cell area at time *t* (*A*_*t*_) to baseline area (*A*_0_), defined as the mean of the first three frames under isotonic conditions. Relative cell volume (*Vol*) was estimated using the spherical approximation:

$$Vol=\frac{4}{3\sqrt{\pi }}*\;A^{\frac{3}{2}}\text{.}$$

To quantify water permeability *P*_*w*_, near-spherical U87-MG cells were exposed to a 30% hypotonic solution (osmolarity reduced from 300 to 210 mOsm), and the resulting volume increase was tracked. The initial slope of normalized volume change (*Vol*/*Vol*_0_) during swelling was used to calculate *P*_*w*_ following established procedures (26).

To assess the contributions of IK, BK, and VRAC channels to volume regulation, near-spherical cells were superfused with isotonic Ringer’s solution containing TRAM-34 (IK channel blocker, 3 μM), paxilline (BK channel blocker, 1 μM), or DCPIB (VRAC blocker, 10 μM) at saturating concentrations determined from prior dose-response studies (10,27,28). To evaluate the impact of low extracellular Na^+^ on cell volume, cells were superfused with isosmotic low-Na^+^ Ringer solutions in which Na^+^ was partially or completely replaced by choline^+^. Projected cell area was measured every 20 s for 35 min and converted to volume using the spherical approximation. Relative volume changes (*Vol*/*Vol*_0_) were used to evaluate the impact of selective channel blockade.

#### Reagents

Amphotericin B, DCPIB, TRAM-34, paxilline, and ouabain were dissolved in DMSO to prepare stock solutions at 50, 10, 6, 10, and 10 mM, respectively. Final working concentrations were 200 μM (amphotericin B), 10 μM (DCPIB), 3 μM (TRAM-34), 1 μM (paxilline), and 5 μM (ouabain). The maximum DMSO concentration in recording solutions was 0.1%, which did not affect membrane currents (data not shown). All reagents were freshly diluted to final concentrations on the day of use and applied via a gravity-driven perfusion system for both patch-clamp and video imaging experiments.

## Results

To illustrate the validity of the proposed inverse method, we constructed a P-L model for the U87-MG GBM cell line. Our analysis proceeded in four steps: 1) determination of the steady-state variables, with emphasis on resting *V*_*m*_ and cell volume, adopting literature values when available (see Table 1); 2) experimental measurement of a subset of P-L parameters, selected based on their accessibility using electrophysiological and imaging techniques available in our laboratory; 3) application of the LP method to infer the remaining, experimentally inaccessible P-L parameters; and 4) comparison of model predictions with experimental data on cell volume regulation obtained in our laboratory.

### Determination of a subset of steady-state variables in U87-MG cells

#### Assessment of resting *V*_*m*_

**Figure 2:**
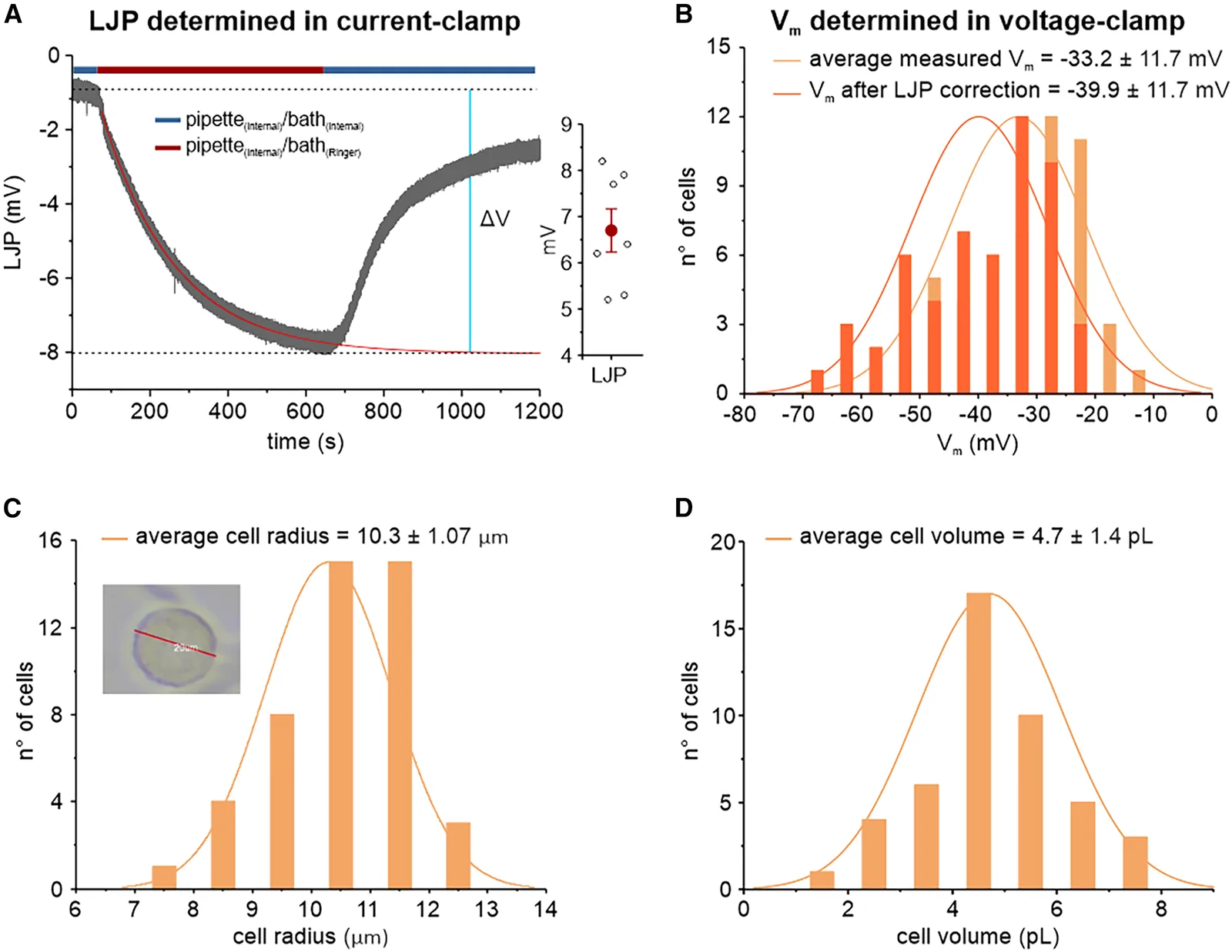
Measurement of resting membrane potential (*V*_*m*_) and cell volume in U87-MG cells using whole-cell current-clamp recordings and live-cell imaging. (*A*) Representative voltage trace showing the measurement of liquid junction potential (LJP). When both pipette and bath contained internal solution, the recorded potential was 0 mV. Perfusion of the bath with external Ringer’s solution induced a hyperpolarizing shift, reflecting the development of the LJP. Replacing the bath with internal solution restored the baseline. The trace was fitted with a monoexponential decay function (*red line*), and the asymptotic value of the fit was taken as the steady-state LJP. The LJP was calculated as the voltage difference (Δ*V* = *V*_*bath*_ − *V*_*pipette*_), with positive values representing the true LJP. Inset: scatter plot of individual LJP measurements (*n* = 7 independent preparations of internal and Ringer solutions). Data are shown as mean ± SEM. The average LJP value was then used to correct the resting *V*_*m*_ recorded in U87-MG cells by subtraction. (*B*) Histogram of resting *V*_*m*_ values recorded from U87-MG cells before (*light orange*) and after (*dark orange*) correction for the LJP. Mean ± SD for each distribution are reported. *V*_*m*_ values were used as steady-state inputs for the P-L model. Corrected *V*_*m*_ values were used as steady-state inputs for the P-L model. (*C*) Histogram of cell radius values (μm), calculated from diameters of rounded, near-spherical U87-MG cells obtained after trypsinization and imaged by live phase-contrast microscopy (*inset*; scale bar, 20 μm). This panel illustrates the limited variability in cell size and supports the validity of the spherical approximation used for volume estimation. (*D*) Histogram of cell volume values (pL), derived from cell radius assuming spherical geometry. Mean ± SD for both cell radius (*C*) and cell volume (*D*) distributions are reported.

To determine the resting *V*_*m*_, a key steady-state variable in the P-L model, we performed whole-cell patch-clamp current-clamp recordings in U87-MG cells. Accurate measurement of *V*_*m*_ requires correction for the liquid junction potential (LJP) arising from ionic differences between the pipette internal solution and the extracellular Ringer’s solution. The LJP was quantified using a standard protocol: the pipette, filled with internal solution, was initially immersed in a recording chamber containing the same solution. Replacement of the bath with Ringer’s solution caused a reproducible hyperpolarizing voltage shift, reflecting differences in ionic mobilities across the junction. In most recordings, the voltage reached a clear plateau during Ringer’s perfusion, corresponding to the steady-state LJP. To ensure robust estimation, even when full recovery upon reperfusion with internal solution was not observed, all voltage traces were fitted with a monoexponential function, and the asymptotic value was taken as the steady-state LJP (Fig. 2 *A*). Across seven independent experiments with freshly prepared solutions, the mean LJP was +6.7 ± 0.5 mV (mean ± SEM; Fig. 2 *A*, inset). After LJP correction, resting *V*_*m*_ under whole-cell current-clamp conditions shifted from −33.2 ± 11.7 mV to −39.9 ± 11.7 mV (Fig. 2 *B*).

#### Assessment of resting cell volume

In parallel, resting cell volume, another key steady-state variable for the P-L model, was quantified under the same experimental conditions. U87-MG cells were imaged 10–15 min after trypsinization and replating, when they adopt a near-spherical morphology suitable for volumetric analysis. Cell diameters were measured from images obtained with live phase-contrast microscopy (Fig. 2 *C*, inset), and volumes were calculated assuming spherical geometry. The average cell radius was 10.3 ± 1.1 μm, corresponding to a mean volume of 4.7 ± 1.4 pL (mean ± SD).

### Determination of a subset of P-L model parameters in U87-MG cells

Among the key parameters of the P-L model for U87-MG cells, we focused on those experimentally accessible. These include the resting conductances for Na^+^ (*gNa*), K^+^ (*gK*), and Cl^−^ (*gCl*) (Figs. 4 and 5), plasma membrane water permeability coefficient (*P*_*w*_) (Fig. 6), and both the maximal transport rate and Na^+^ affinity of the Na^+^/K^+^-ATPase pump (Fig. 7). Although the P-L model also includes parameters that cannot be directly measured, we demonstrate that the experimentally accessible subset is sufficient to determine the remaining parameters using the inverse method described herein.

#### Assessment of resting Na^+^, K^+^, and Cl^−^ conductance (*gNa*, *gK,* and *gCl*)

Before estimating individual conductances, it is important to note that both the resting K^+^ *(gK)* and Cl^−^ conductance *(gCl)* arise from the combined activity of multiple ion channels. In U87-MG cells, *gK* includes not only the leak K^+^ conductance ($g_{K}^{L}$) but also small yet significant contributions from intermediate-conductance (IK, $g_{K}^{IK}$) and large-conductance (BK, $g_{K}^{BK}$) Ca^2+^-activated K^+^ channels (11). Accordingly, the total resting K^+^ conductance can be expressed as

$$g_{K}=g_{K}^{L}+g_{K}^{IK}+g_{K}^{BK}$$

Similarly, the resting Cl^−^ conductance *(gCl)* comprises a leak component $g_{Cl}^{L}$ and the resting activity of VRAC $g_{Cl}^{VRAC}$ (29):

$$g_{Cl}=g_{Cl}^{L}+g_{Cl}^{VRAC}$$

To determine the total resting currents for these ions, we quantify not only the leak conductances but also the specific contributions of $g_{K}^{IK}$, $g_{K}^{BK}$, and $g_{Cl}^{VRAC}$.

#### Assessment of leak *g*_*Na*_, *g*_*K*_*,* and *g*_*Cl*_

**Figure 3:**
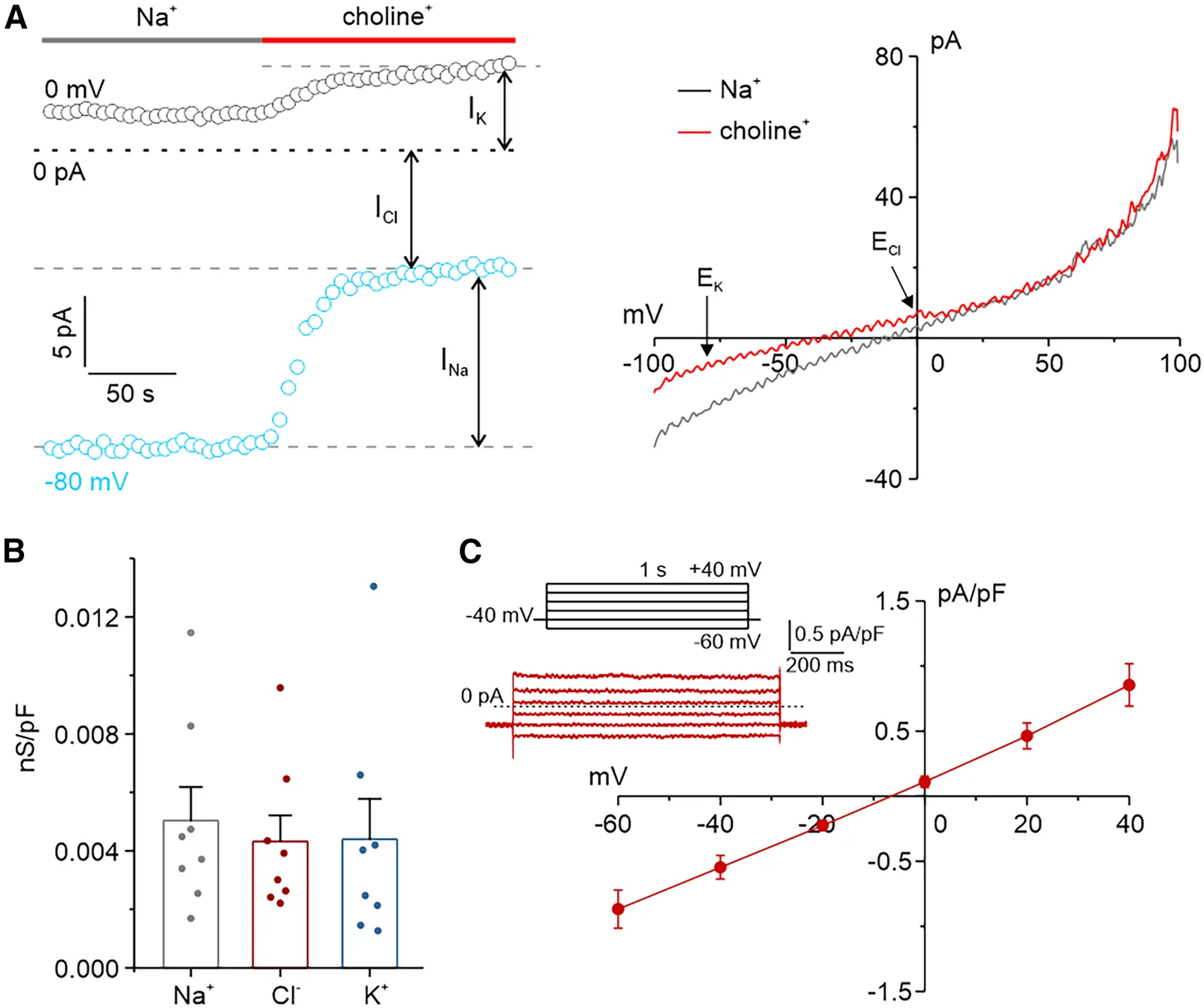
Electrophysiological assessment of leak *g*_*Na*_*, g*_*K*_*,* and *g*_*Cl*_ in U87-MG cells. (*A*) *Left:* Representative time courses of whole-cell current (pA) measured at 0 mV (*top, gray circles*) and −80 mV (*bottom, cyan circles*) during the voltage ramps. *Right:* Representative current traces (pA) recorded in response to 1-s voltage ramps from −100 to +100 mV (holding potential: −40 mV), using either standard extracellular Ringer's solution containing Na^+^ (*gray trace*) or a modified solution in which Na^+^ was replaced with the impermeant cation choline^+^ (*red trace*). Arrows indicate the reversal potentials for K^+^ (E_K_ = −80 mV) and Cl^−^ (E_Cl_ = 0 mV) under the recording conditions. (*B*) Bar graph showing average leak conductance densities (nS/pF). Conductances were derived from current amplitudes at the two reversal potentials: *gNa* was calculated at E_K_ by subtracting the residual current recorded in choline^+^ from that in standard Ringer's solution; *gCl* and *gK* were estimated as the residual current in choline-containing solution at E_K_ and E_Cl_, respectively. Data are shown as mean ± SEM; *n* = 8 cells from three independent cultures. (*C*) Average current-voltage (I-V) relationships recorded in U87-MG cells (*n* = 13 cells from three independent cultures). Currents were elicited by 1-s depolarizing voltage steps from −60 to +40 mV in 20-mV increments, from a holding potential of −40 mV. *Inset*: representative current traces from a single cell illustrating the voltage-step protocol (*drawn above the traces*). Data are shown as mean ± SEM.

Leak conductances $g_{Na}^{L}$, $g_{K}^{L}$, and $g_{Cl}^{L}$ were quantified using 1-s voltage ramps from −100 mV to +100 mV. Whole-cell voltage-clamp recordings were performed while pharmacologically blocking all major active channels (BK, IK, and VRAC) using 1 μM paxilline, 3 μM TRAM-34, and 10 μM DCPIB, respectively (10,11,12,29). Recordings were performed in the absence of intracellular ATP to suppress Na^+^/K^+^-ATPase activity, thereby isolating the purely passive leak Na^+^, K^+^, and Cl^−^ currents. To separate ion-specific contributions, extracellular Na^+^ was replaced with impermeant choline^+^ (Fig. 3 *A*). The current measured at 0 mV, corresponding to the Cl^−^ reversal potential (*E*_*Cl*_), in choline^+^-substituted solution was attributed to K^+^ and used to calculate $g_{K}^{L}$. Conversely, the current at the K^+^ reversal potential (*E*_*K*_) was attributed to Cl^−^ and used to determine $g_{Cl}^{L}$. The Na^+^ leak conductance ($g_{Na}^{L}$) was derived from the difference in currents at both reversal potentials before and after Na^+^ substitution, assuming a linear (ohmic) current-voltage (I-V) relationship. All conductances were normalized to the membrane capacitance of individual cells and are summarized in Fig. 3 *B*.

To validate the form of the P-L model used, we assessed whether leak conductances in U87-MG cells are time- and voltage-independent within the physiological voltage range. A series of 1-s voltage steps from −60 mV to +40 mV produced currents that exhibited no detectable time dependence and displayed a linear I-V relationship without inward or outward rectification (Fig. 3 *C*). These findings confirm that passive conductances in U87-MG cells can be treated as time- and voltage-independent, supporting their use as linear parameters in the P-L model.

**Figure 4:**
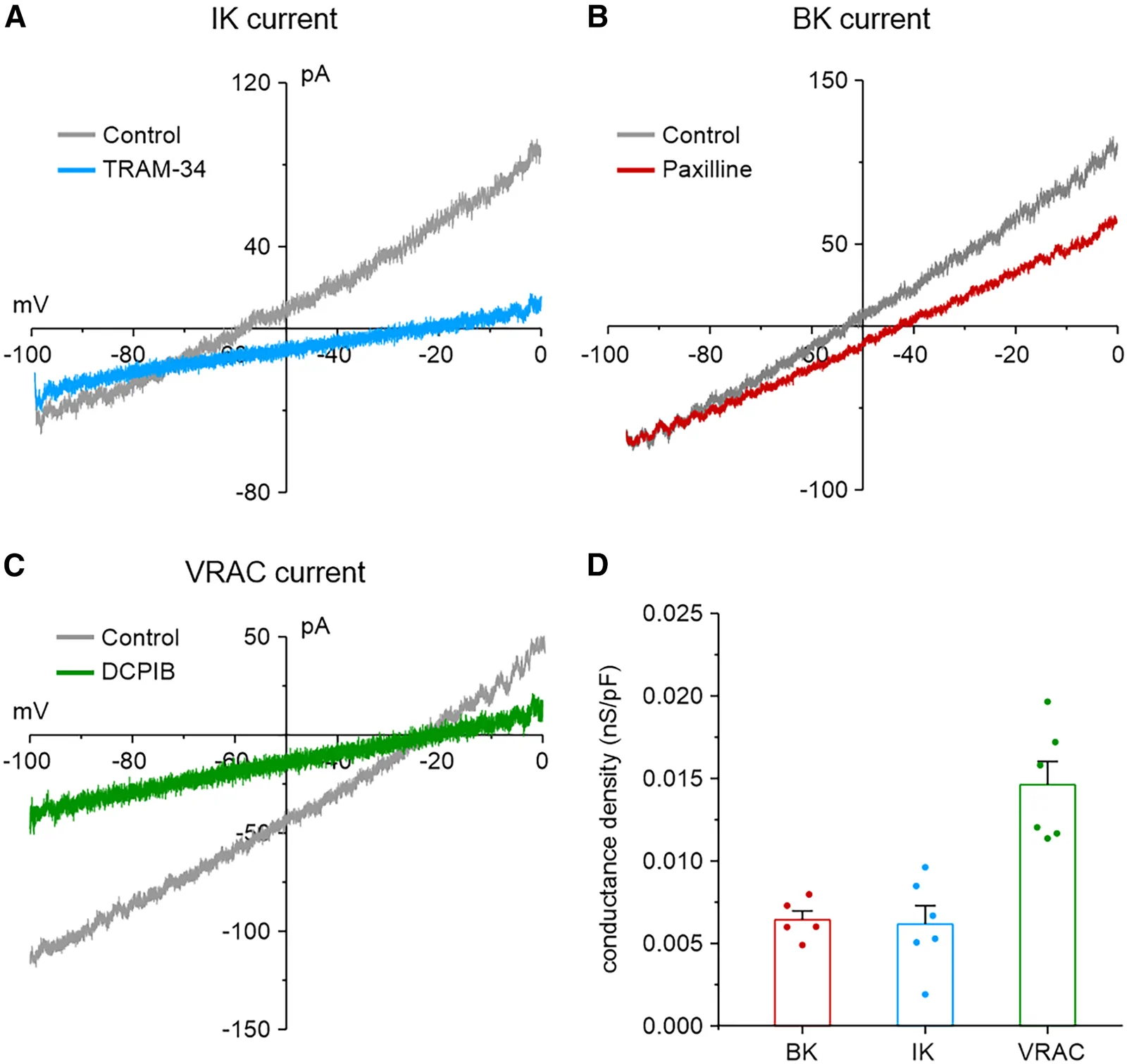
Electrophysiological assessment of resting IK, BK, and VRAC conductances in U87-MG cells. (*A*–*C*) Representative whole-cell current traces (pA) recorded in response to 1-s voltage ramps from −100 to 0 mV (holding potential: −40 mV), under control conditions (*light gray traces*) and after application of selective channel inhibitors: 3 μM TRAM-34 (*A, cyan trace*) for IK channels, 1 μM paxilline (*B, red trace*) for BK channels, and 10 μM DCPIB (*C, green trace*) for VRAC. (*D*) Bar graph showing average conductance densities (nS/pF) calculated from current amplitudes at −40 mV, using the corresponding ion reversal potentials for K^+^ (IK, *n* = 6 cells, and BK, *n* = 5 cells) and Cl^−^ (VRAC, *n* = *5* cells) based on the patch-clamp recording conditions. Data are shown mean ± SEM. All experiments were replicated in at least three independent cultures.

*IK, BK, and VRAC conductance under resting conditions.* In addition to leak currents, IK, BK, and VRAC channels contribute to the resting membrane conductance of U87-MG cells. To quantify the basal activity of each channel, perforated whole-cell patch-clamp recordings were performed to preserve endogenous intracellular Ca^2+^ levels and impermeant anions, conditions essential for physiological gating of IK, BK, and VRAC channels. Cells were voltage-clamped with 1-s voltage ramps from −100 mV to 0 mV, with a holding potential of −40 mV. Selective pharmacological blockers were applied to isolate each channel contribution: 3 μM TRAM-34 for IK, 1 μM paxilline for BK, and 10 μM DCPIB for VRAC. Basal channel activity was determined by subtracting blocker-insensitive currents from control traces, revealing the basal current attributable to each respective conductance (Fig. 4, *A*–*C*). Current amplitudes at −40 mV were converted to conductance densities (nS/pF) using established reversal potentials for K^+^ (IK and BK) and Cl^−^ (VRAC). Quantitative analysis confirmed that all three channels exhibit significant activity at rest (Fig. 4 *D*; Table 3).

#### Assessment of *P*_*w*_ via hypotonic-induced cell swelling

**Figure 5:**
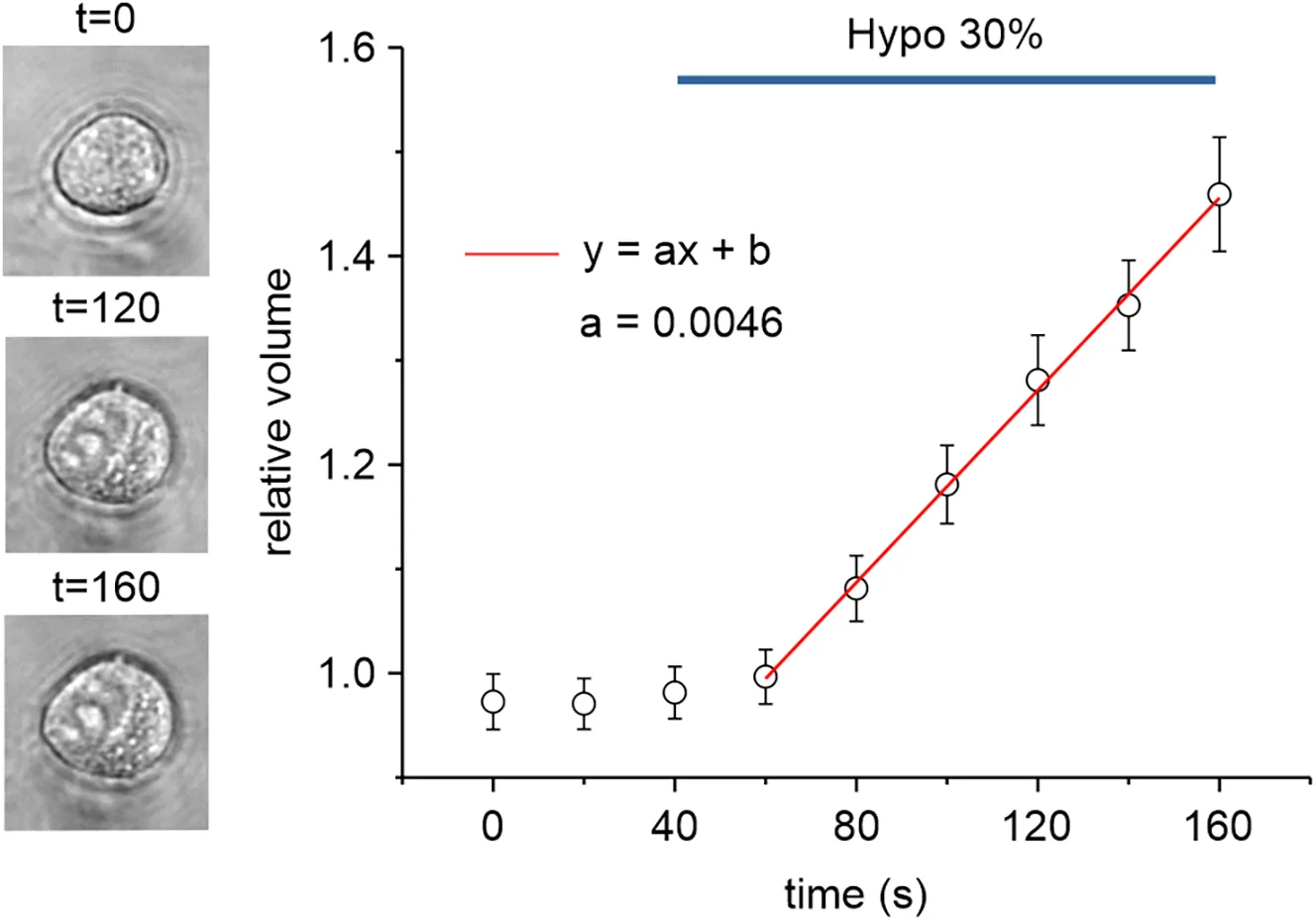
Assessment of cell volume changes to evaluate plasma membrane water permeability (*P*_*w*_) in U87-MG cells. *Left*: Representative phase-contrast images of spherical U87-MG cells acquired under isotonic conditions (t = 0 s) and at two time points during exposure to a 30% hypotonic solution (t = 120 s and t = 160 s). *Right*: Time course of relative cell volume changes before and during application of the hypotonic solution (*indicated by the dark blue bar*). The first three data points represent baseline measurements under isotonic conditions. The initial rate of swelling during the first 120 s of hypotonic exposure was fitted with a linear function (*y = ax + b*), where the slope (*a*) was used as an index of plasma membrane water permeability (P_w_). Data are shown as mean ± SEM; *n* = 8 cells from three independent cultures.

To quantify *P*_*w*_ in U87-MG cells, we exposed near-spherical cells to a 30% hypotonic solution and monitored volume changes using phase-contrast microscopy. Images were acquired every 20 s for 160 s, and cell volumes were estimated by tracking the projected cell area and assuming spherical geometry. To reduce variability due to cell size heterogeneity, only cells with projected areas within ±10% of the mean were analyzed (see materials and methods). Under isotonic conditions, cell volume remained stable, confirming baseline homeostasis. Upon hypotonic challenge, cells exhibited a rapid, approximately linear increase in volume during the initial 120 s, consistent with osmotic water influx (Fig. 5). The initial swelling rate was determined by linear regression of normalized volume (*Vol*/*Vol*_0_) over this interval and used to estimate *P*_*w*_, as previously described (26), yielding a rate of 0.0046/s. This corresponds to a volumetric water influx of ∼1.93 × 10^−17^ m^3^/s per cell, assuming an average baseline volume of 4.2 × 10^−15^ m^3^ (Fig. 2 *C*). Given the imposed osmotic gradient of 90 mOsm (from 300 to 210 mOsm), this rate corresponds to a calculated *P*_*w*_ of 2.14 × 10^−16^ m^3^/(s^∗^M) (Fig. 5).

This approach directly quantifies the volumetric water flux driven by the osmotic gradient, rather than normalizing flux to membrane area. Expressing water permeability as *P*_*w*_ allows cell water permeability to be considered independent of cell swelling, consistent with the assumptions of the P-L model.

For comparison with classical measurements, the osmotic permeability coefficient *P*_*f*_ (in cm/s), as defined by Finkelstein (30), can be derived from *P*_*w*_ using

$$P_{f}=\frac{P_{w}}{A\cdot V_{w}}\text{,}$$

where *A* is the cell surface area, and *V*_*w*_ is the molar volume of water (∼18 × 10^−6^ m^3^/mol). Assuming an average cell radius of 10 μm (Fig. 2 *C*), our measured *P*_*w*_ corresponds to *P*_*f*_ = 9.4 × 10^−4^ cm/s, a value well within the range reported for mammalian cells (10^−4^–10^−2^ cm/s) (31,32).

#### Assessment of Na^+^/K^+^-ATPase activity and intracellular Na^+^ sensitivity

**Figure 6:**
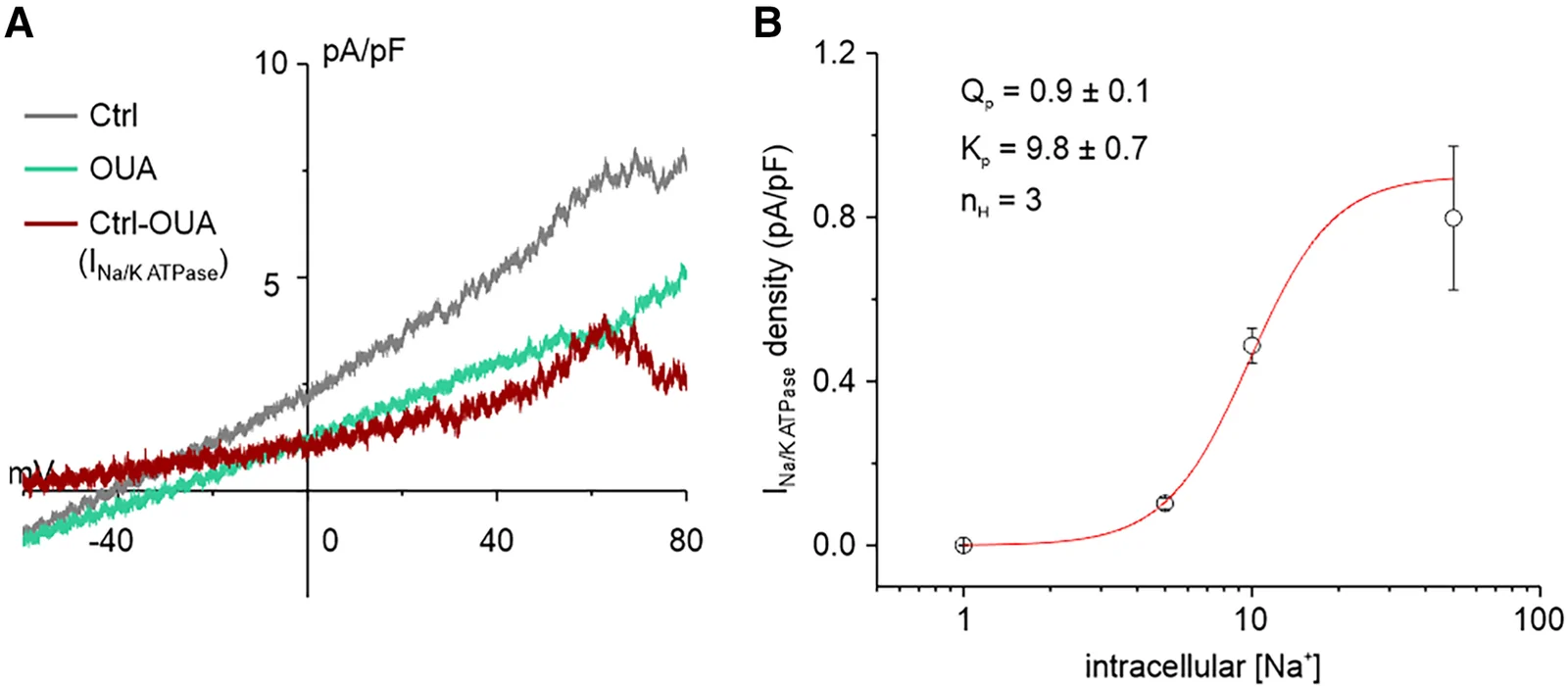
Electrophysiological analysis of Na^+^/K^+^-ATPase activity in U87-MG cells. (*A*) Representative current density traces (pA/pF) recorded from U87-MG cells using 1-s voltage ramps from −60 to +80 mV (holding potential: −40 mV), under control conditions (Ctrl, *gray trace*) and during application of 5 μM ouabain (OUA, *green trace*). The ouabain-sensitive current, representing the Na^+^/K^+^ ATPase current (I_Na/K ATPase_) and obtained by subtracting the ouabain trace from the control trace, is shown on the same I-V axes (Ctrl-OUA, *red trace*). Recordings were performed using an internal solution containing 50 mM Na^+^ to stimulate Na^+^/K^+^ ATPase activity. (*B*) Semilogarithmic dose-response curve of I_Na/K ATPase_ density at 0 mV, plotted as a function of intracellular Na^+^ concentration (1–50 mM). Current amplitude was calculated as the difference between baseline and ouabain-insensitive current at each Na^+^ concentration. The solid line represents a fit to the Hill equation: I_Na/K ATPase_$=Q_{p}/\left(1+K_{p}/{\left[{Na}^{+}\right]}^{3}\right)$. Best fit parameters: Q_p_ = 0.9 ± 0.1 pA/pF; K_p_ = 9.8 ± 0.7 mM. Data are shown as mean ± SEM; three to four cells per condition. All experiments were replicated in at least three independent cultures.

To quantify the electrogenic activity of the Na^+^/K^+^-ATPase and its dependence on intracellular Na^+^ concentration, perforated whole-cell patch-clamp recordings were performed, preserving endogenous ATP levels required for physiological pump function. Whole-cell currents were elicited using 1-s voltage ramps from −60 to +80 mV with a holding potential of −40 mV. Pump-mediated currents were isolated by subtracting traces recorded in the presence of 5 μM ouabain from control recordings. The resulting ouabain-sensitive current is plotted alongside the control and ouabain traces for direct comparison (Fig. 6 *A*). The ouabain-sensitive current displayed a near-linear relationship with membrane voltage, consistent with modest voltage dependence reported for the Na^+^/K^+^-ATPase (33,34).

To examine the dependence of the Na^+^/K^+^-ATPase activity on intracellular Na^+^, perforated patch-clamp recordings were performed using internal solutions containing defined Na^+^ concentrations (1–50 mM). At 0 mV, the amplitude of the ouabain-sensitive current increased as a function of intracellular Na^+^ (Fig. 6 *B*). Fitting data with the Hill equation (Hill coefficient, n_H_ = 3), yielded a dissociation constant (*k*_*p*_) of 9.8 ± 0.7 mM and a maximal pump current density (*Q*_*p*_) of 0.9 ± 0.1 pA/pF (mean ± SEM, *n* = 3–4 cells per condition). The measured Na^+^ affinity (*k*_*p*_ ≈ 9.8 mM) is in excellent agreement with values reported for mammalian Na^+^/K^+^-ATPase. Zahler et al. (35) reported ∼12 mM for the α1 isoform in HeLa cells, and Brodsky and Guidotti (36) found comparable values (∼9–12 mM) in brain synaptosomal preparations. Finally, assuming a turnover rate of ∼100 cycles/s per pump molecule, the maximal pump current density corresponds to an estimated Na^+^/K^+^-ATPase surface density of ≈560 pumps/μm^2^. This value lies within the physiological range of 400–1200 pumps/μm^2^ reported for mammalian cells (34,37,38,39). These results confirm that the ouabain-sensitive current reflects a realistic and physiologically relevant membrane density of Na^+^/K^+^-ATPase molecules in U87-MG cells.

### Application of the LP method to determine the full set of P-L model parameters

The general LP framework described above was applied to estimate the complete set of P-L model parameters for the U87-MG GBM cell line. Although the preceding LP section established the mathematical formulation and iterative structure, here, we demonstrate its application to experimental data to resolve the six unknown steady-state parameters (*g*_*Na*_, *g*_*K*_, *g*_*Cl*_, *Q*_*p*_, *Q*_*NKCC*_, *Q*_*KCC*_). Table 4 summarizes the results obtained using the custom LP implementation provided in the supporting material.

**Table 4:** Sequential LP analysis of the P-L model for U87-MG GBM cells under resting conditions considering different numbers of fixed parameters

No parameters fixed	0 ≤ **g**_Na_ ≤ **∞** 0 ≤ **g**_**K**_ ≤ **∞** 0 ≤ **g**_Cl_ ≤ **∞** 0≤ **Q**_**p**_ ≤ **∞** 0 ≤ **Q**_NKCC_ ≤ **∞** 0 ≤ **Q**_KCC_ ≤ **∞**
*g*_*Na*_fixed to 17.78 *nS*/*M*	${17.78\leq g}_{Na}\leq 17.78\frac{\text{nS}}{M}$ 0 ≤ *g*_*K*_ ≤ *∞*$\frac{\text{nS}}{M}$ ${0\leq g}_{Cl}\leq \infty \frac{\text{nS}}{M}$ $1.29{\times 10}^{-4}\leq Q_{p}\leq \infty \frac{pmol}{s}$ $0{\leq Q}_{NKCC}\leq \infty \frac{pmol}{s\;M^{4}}$ $0\leq Q_{KCC}\leq \infty \frac{pmol}{s\;M^{2}}$
*g*_*Na*_ fixed to 17.78*nS*/*M* *g*_*K*_ fixed to 3.75 *nS*/*M*	${17.78\leq g}_{Na}\leq 17.78\frac{\text{nS}}{M}$ $3.75\leq g_{K}\leq 3.75\frac{\text{nS}}{M}$ ${4.47\leq g}_{Cl}\leq \infty \frac{\text{nS}}{M}$ $1.74{\times 10}^{-4}\leq Q_{p}\leq \infty \frac{pmol}{s}$ $29.7{\leq Q}_{NKCC}\leq \infty \frac{pmol}{s\;M^{4}}$ $0\leq Q_{KCC}\leq \infty \frac{pmol}{s\;M^{2}}$
*g*_*Na*_ fixed to 17.78 *nS*/*M* *g*_*K*_ fixed to 3.75 *nS*/*M* *g*_*Cl*_ fixed to 4.52 *nS*/*M*	${17.78\leq g}_{Na}\leq 17.78\frac{\text{nS}}{M}$ $3.75\leq g_{K}\leq 3.75\frac{\text{nS}}{M}$ ${4.52\leq g}_{Cl}\leq 4.52\;\frac{\text{nS}}{M}$ 1.77 × 10^−4^≤*Q*_*p*_ ≤ 1.77 × 10^−4^$\frac{pmol}{s}$ $31.6{\leq Q}_{NKCC}\leq 31.6\frac{pmol}{s\;M^{4}}$ $5.54{\times 10}^{-4}\leq Q_{KCC}\leq 5.54{*10}^{-4}\frac{pmol}{s\;M^{2}}$

The analysis proceeds stepwise, illustrating how successive incorporation of experimentally measured conductances transforms an initially underdetermined system into a unique, self-consistent solution. When all six parameters were left unconstrained, both the upper and lower bounds were unbounded for each parameter, confirming that the steady-state flux equations alone do not uniquely determine the conductances and fluxes; any positive values satisfy the equations. Introducing the first experimental constraint, *g*_*Na*_ = 17.78 nS/M, defined an inward Na^+^ leak. Steady-state Na^+^ balance (Eq. 11) then required that this inward Na^+^ flux, combined with the inward Na^+^ flux produced by the NKCC cotransporter (*Q*_*NKCC*_), be counterbalanced by outward active transport through the Na^+^/K^+^-ATPase (*Q*_*p*_). This introduces a “minimum required activity” for *Q*_*p*_ to maintain the Na^+^ concentration at equilibrium, yielding nonzero “lower bound” for this parameter. (*Q*_*p*_ ≥ 1.29 × 10^−4^ pmol/s). Because larger pump fluxes do not violate the steady-state constraints, no upper bound was imposed. When *g*_*K*_ = 3.75 nS/M was additionally fixed, the magnitude of K^+^ efflux became similarly constrained. Maintaining the resting *V*_*m*_ then required minimal values of Cl^−^ permeability (*g*_*Cl*_) and Na^+^/K^+^ ATPase activity (*Q*_*p*_); parameters below these thresholds violated charge- and ion-balance constraints, yielding one-sided lower bounds (*g*_*Cl*_ ≥ 4.47 nS/M and *Q*_*p*_ ≥ 1.74 × 10^−4^ pmol/s). Finally, constraining *g*_*Cl*_ to its experimental value (4.52 nS/M), collapsed the feasible region to a single, physically consistent steady-state solution. With all three conductances specified, the electroneutrality constraint (Eq. 14) imposed a unique value for Na^+^/K^+^-ATPase activity (*Q*_*p*_). With *g*_Na_ and *Q*_*p*_ fixed, the Na^+^ balance equation (Eq. 11) then determined the rate of the NKCC cotransporter (*Q*_*NKCC*_), and the K^+^ and Cl^−^ balance equations (Eqs. 12 and 13) fixed the KCl cotransporter flux (*Q*_*KCC*_). The resulting parameter set was as follows: *Q*_*p*_ = 1.77 × 10^−4^ pmol/s; *Q*_*NKCC*_ = 31.6 pmol/(s^∗^M^4^); and *Q*_*KCC*_ = 5.54 × 10^−4^ pmol/(s^∗^M^2^). Thus, starting from six linear unknown transport parameters, the LP approach resolves three of them once the three passive conductances are experimentally constrained, producing a complete steady-state solution consistent with both the P-L model and measured electrophysiological properties.

Notably, the LP-derived value of *Q*_*p*_ (1.77 × 10^−4^ pmol/s) is approximately half the maximal Na^+^/K^+^-ATPase rate measured electrophysiologically at 0 mV (0.9 ± 0.1 pA/pF, corresponding to ≈3.35 3.35 × 10^−4^ pmol/s considering a membrane capacitance of 36 pF). This discrepancy likely reflects the voltage dependence of the Na^+^/K^+^-ATPase, as the LP-derived value corresponds to the resting *V*_*m*_ (≈−40 mV) rather than 0 mV. Importantly, the LP-derived *Q*_*p*_ value emerges independently of experimental pump measurements, underscoring the internal consistency and predictive validity of the inverse LP estimation.

### Experimental validation of the LP method

#### Selective blockade of IK, BK, and VRAC channels induces progressive cell swelling in U87-MG cells

**Figure 7:**
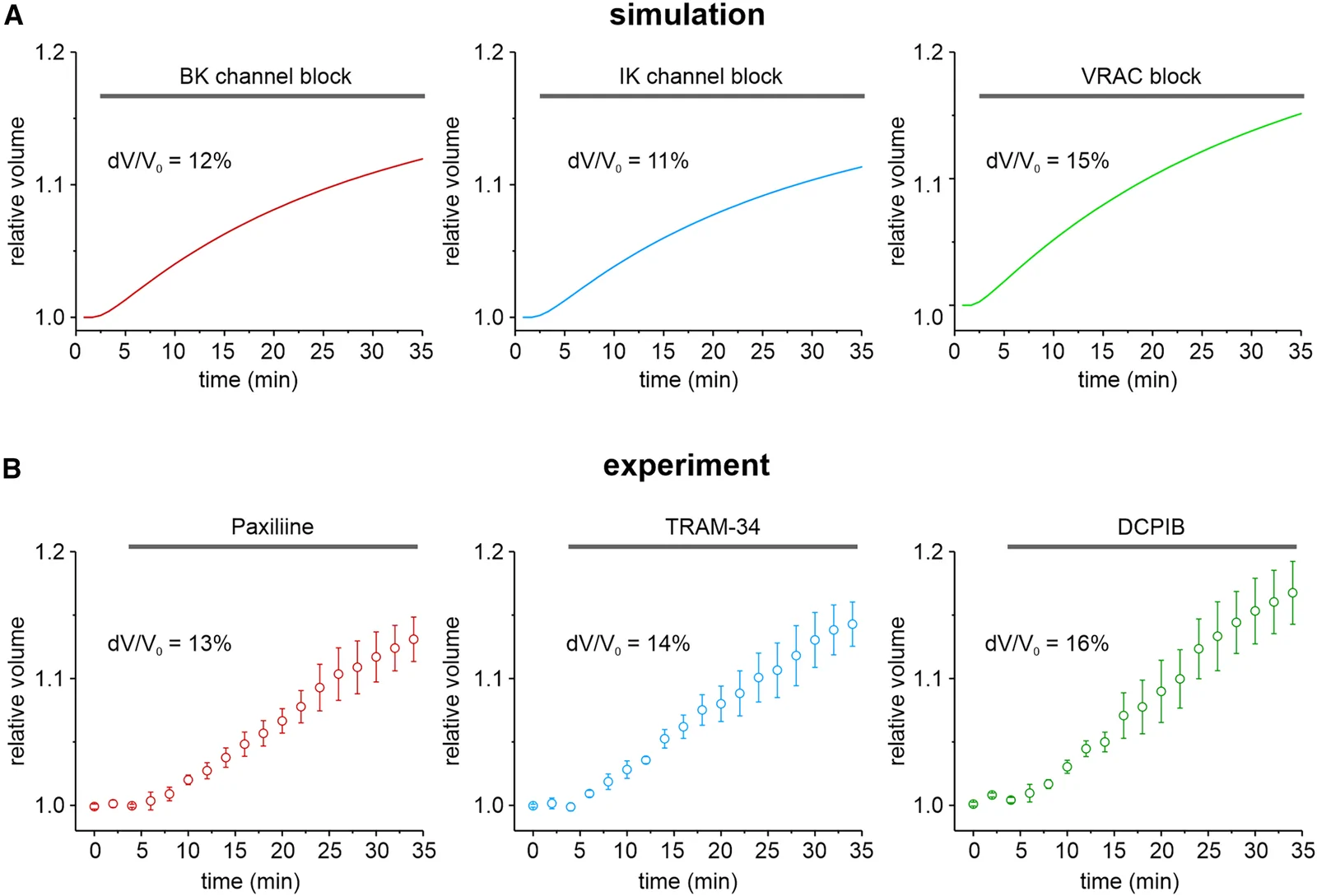
Simulated and experimental analysis of cell volume changes in U87-MG cells after selective blockade of IK, BK, and VRAC channels. (*A*) Simulated time courses of relative cell volume changes using the P-L model under selective inhibition of BK (*left, red trace*), IK (*middle, cyan trace*), and VRAC (*right, green trace*) channels. In all cases, the model predicts a gradual increase in cell volume over time. (*B*) Experimentally measured relative cell volume changes, assessed by live-cell video imaging of spherical U87-MG cells. Selective pharmacological inhibition of IK, BK, and VRAC channels was achieved using 3 μM TRAM-34 (*left, cyan circles*; *n* = 4 cells), 1 μM paxilline (*middle, red circles*; *n* = 4 cells), and 10 μM DCPIB (*right, green circles*; *n* = 4 cells), respectively. Projected cell area was measured and converted to relative volume using the spherical approximation described in the materials and methods. Experimental results closely matched model predictions, showing a slow, progressive increase in cell volume. Data are shown as mean ± SEM. All experiments were replicated in at least three independent cultures.

To validate the inverse approach used for parameter estimation and assess the physiological relevance of the P-L model, we examined the contribution of IK, BK, or VRAC channels to volume regulation by comparing computational predictions and experimental data obtained during pharmacological inhibition of IK, BK, and VRAC channels. Simulations based on the fully parameterized P-L model predicted that selective blockade of each channel would cause a gradual increase in relative cell volume. After 35 min, relative volume increases were 11% for IK inhibition, 12% for BK inhibition, and 15% for VRAC inhibition (Fig. 7 *A*). Although the magnitude of swelling differed modestly among the three conditions, the overall kinetics were qualitatively similar. These predictions are consistent with disruption of steady-state ion fluxes, intracellular accumulation of osmotically active solutes, and consequent passive water influx in the absence of compensatory volume-regulatory mechanisms.

To experimentally test these predictions, volume changes were quantified in near-spherical U87-MG cells using time-lapse video imaging after selective channel blockade. Cells were treated with 3 μM TRAM-34 (IK channel blocker), 1 μM paxilline (BK channel blocker), or 10 μM DCPIB (VRAC blocker), and projected cell areas were converted to volumes using spherical approximation (see materials and methods). Consistent with model predictions, all three treatments induced a slow and sustained increase in cell volume over 35 min (Fig. 7 *B*). Final volume increases were 14% for IK inhibition, 13% for BK inhibition, and 16% for VRAC inhibition, closely matching the simulated values.

These results confirm that basal activity of IK, BK, and VRAC channels is critical for maintaining osmotic and volume homeostasis in U87-MG cells. The strong agreement between simulated and experimental outcomes supports the accuracy of the P-L model parameters as determined via the LP-based inverse method.

**Figure 8:**
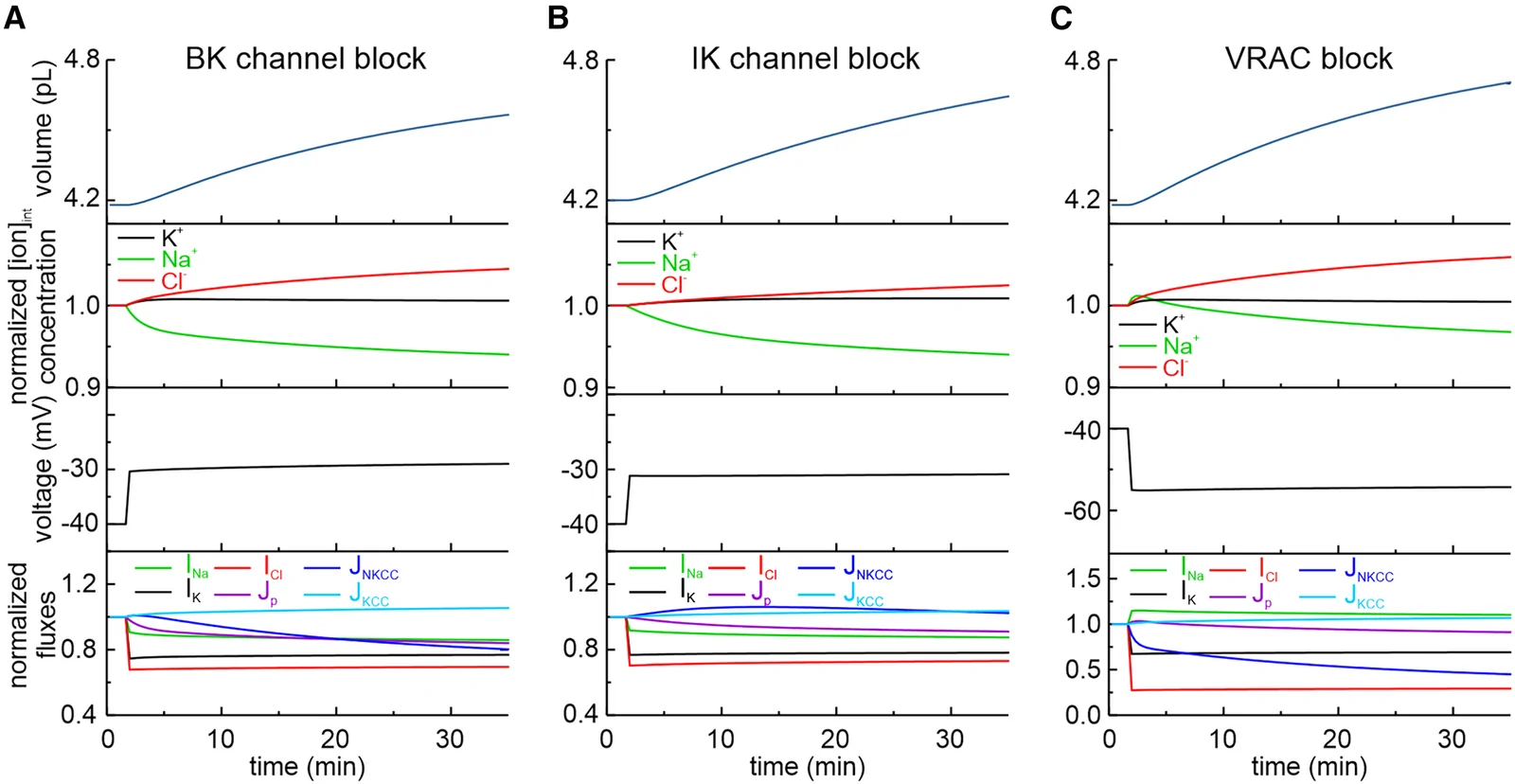
Model-predicted mechanisms of cell swelling after selective blockade of K^+^ and Cl^−^ conductances. (*A*–*C*) Simulated time courses showing the dynamic response of U87-MG cells to pharmacological inhibition of BK (*A*), IK (*B*), and VRAC (*C*) channels. For each condition, the panels display: relative cell volume (*top row*), intracellular Na^+^, K^+^, and Cl^−^ concentrations normalized to baseline (*second row*), *V*_*m*_ (mV; *third row*), and major ion fluxes (I_Na_, I_K_, I_Cl_, Na^+^/K^+^-ATPase (J_Q_), NKCC, and KCC cotransport; normalized to t = 0; *bottom row*). Blockade of IK or BK channels induces membrane depolarization, suppresses Cl^−^ efflux, and promotes intracellular accumulation of K^+^ and Cl^−^, resulting in osmotic water influx and progressive cell swelling. VRAC inhibition leads to membrane hyperpolarization, suppression of K^+^ efflux, and similar osmotic imbalances. In all cases, disruption of ion homeostasis alters Na^+^ influx and secondarily reduces Na^+^/K^+^-ATPase activity, contributing to a slower, sustained component of cell swelling. Together, the simulations highlight both distinct and convergent pathways of volume dysregulation after inhibition of key ion channels.

To gain mechanistic insights into the observed volume changes, we analyzed the time evolution of intracellular ion concentrations, *V*_*m*_, and ion fluxes predicted by the P-L model under the same conditions (Fig. 8, *A*–*C*). After K^+^ channel inhibition (BK or IK), the immediate effect is a membrane depolarization of ∼10 mV. This depolarization diminishes the electrochemical driving force for Cl^−^ efflux, reducing the I_Cl_ component and leading to intracellular Cl^−^ accumulation. Simultaneously, blocked K^+^ efflux causes intracellular K^+^ to rise, further increasing intracellular osmolarity, driving water influx, and promoting cell swelling. Membrane depolarization also reduces the driving force for Na^+^ influx, causing a slight but sustained decrease in I_Na_ and a corresponding reduction of intracellular Na^+^ concentration. Because Na^+^/K^+^-ATPase is Na^+^ dependent, its function declines, reducing net osmolyte efflux and contributing to a slower, secondary phase of cell swelling during the simulation.

In contrast, VRAC inhibition (Fig. 8 *C*) produces a rapid membrane hyperpolarization, substantially reducing the driving force for K^+^ efflux through I_K_, thereby suppressing K^+^ loss. Concurrently, VRAC blockade directly inhibits Cl^−^ efflux, resulting in intracellular accumulation of both ions, elevating osmolarity, and driving water influx. Unlike K^+^ channel inhibition, the membrane hyperpolarization transiently enhances the driving force for Na^+^ entry, producing a brief elevation in intracellular Na^+^. This initial Na^+^ accumulation is subsequently followed by a decline due to suppressed activity of the NKCC transporter, which depends on Na^+^ entry to maintain electroneutral transport. Consequently, the intracellular Na^+^ falls below control levels, attenuating Na^+^/K^+^-ATPase activity and promoting a slower, sustained increase in cell volume, analogous to the secondary swelling phase observed during K^+^ channel blockade.

Together, these simulations highlight distinct yet convergent mechanistic pathways by which K^+^ and Cl^−^ channel blockade disrupt flux balance, leading to progressive intracellular solute accumulation, osmotic water influx, and swelling. The model further demonstrates how *V*_*m*_ changes propagate through coupled ion transport systems, emphasizing the complex interplay governing volume regulation in U87-MG cells.

#### Reduced extracellular Na^+^ leads to progressive cell volume decrease in U87-MG cells

**Figure 9:**
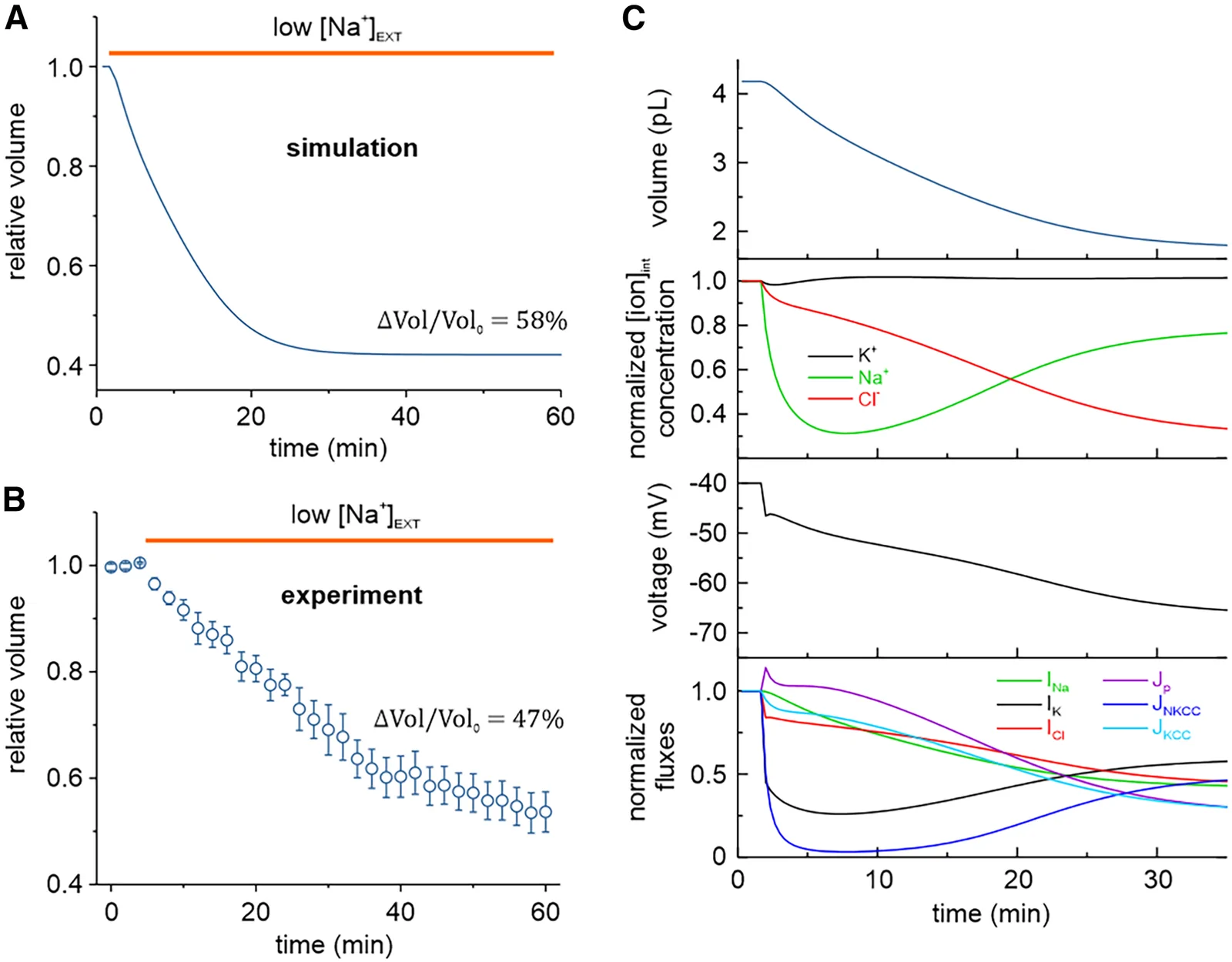
Simulated and experimental analysis of cell volume changes in U87-MG cells under low extracellular Na^+^ conditions and the underlying mechanisms. (*A*) Simulated time course of relative cell volume changes predicted by the P-L model after a reduction in extracellular Na^+^ concentration to 14 mM, with osmolarity maintained constant via substitution with an impermeant monovalent cation. The model predicts a progressive decline in cell volume over time. (*B*) Experimentally measured changes in relative cell volume, assessed in U87-MG cells exposed to low-Na^+^ extracellular solution (14 mM Na^+^ + 126 mM choline^+^). Volume was estimated from phase-contrast microscopy using a spherical approximation of projected cell area described in the materials and methods. Experimental results closely follow the simulation, showing a gradual decrease in cell volume under low-Na^+^ conditions. Data are shown as mean ± SEM (*n* = 6 cell, from three independent cultures). (*C*) Simulated time courses of key variables in the P-L model under reduced extracellular Na^+^: changes in relative cell volume (*top*), intracellular Na^+^, K^+^, and Cl^−^ concentrations (*second row*), *V*_*m*_ (*third row*), and transmembrane ion fluxes (*bottom row*), all normalized to their respective baseline values. The simulation reveals a sequence of ionic and electrical events driving osmotic imbalance and progressive cellular dehydration. ΔVol/Vol_0_ in (*A*) and (*B*) represents the relative change in cell volume with respect to the initial volume (Vol_0_).

To assess the contribution of extracellular Na^+^ to volume regulation, we combined simulations and experiments to examine the response of U87-MG cells to a low-Na^+^ isosmotic extracellular environment. In silico, extracellular Na^+^ concentration was reduced by 90% (from 140 mM to 14 mM) with the removed Na^+^ replaced by an impermeant monovalent to maintain osmolarity. This manipulation produced a gradual decline in relative cell volume over time, consistent with intracellular osmolyte loss and water efflux (Fig. 9 *A*).

Experimentally, U87-MG cells were exposed to a low-Na^+^ extracellular solution containing 14 mM Na^+^, with choline^+^ substituted for Na^+^ to maintain osmotic balance. As predicted, cells exhibited a slow, sustained volume decrease, and the magnitude and kinetics closely matched model predictions (Fig. 9 *B*). Model analysis revealed that shrinkage was initiated by a rapid decrease in intracellular Na^+^ concentration, followed by a slower, sustained reduction in intracellular K^+^ and Cl^−^ (Fig. 9 *C*). This secondary KCl loss reflects diminished Na^+^ influx and consequent suppression of Na^+^/K^+^-ATPase activity, which depends on intracellular Na^+^ for pump cycling (Fig. 6 *B*), together with passive KCl efflux. The resulting decrease in intracellular osmolarity promotes water loss and cell shrinkage. The model further predicts a biphasic hyperpolarization of *V*_*m*_ under low extracellular Na^+^: an initial rapid hyperpolarization due to suppression of Na^+^ leak conductance, followed by a slower hyperpolarization associated with reduced NKCC activity and progressive intracellular Cl^−^ depletion. These findings identify Na^+^ leak and NKCC-mediated Cl^−^ loading as critical determinants of steady-state *V*_*m*_ in U87-MG cells.

Together, these results demonstrate that extracellular Na^+^ availability is a major regulator of cell volume homeostasis. The close agreement between experimental measurements and model predictions further validates the P-L model and underscores the robustness of the LP-based inverse parameter estimation approach.

#### Selective modulation of IK, BK, VRAC, and Na^+^-dependent pathways induces distinct and qualitatively predictable changes in the resting *V*_*m*_ of U87-MG cells

To further evaluate the accuracy of the LP-derived P-L model parameters and extend validation to the electrical domain, we examined how selective perturbation of major ionic pathways influences the resting *V*_*m*_ of U87-MG cells. Simulations predicted that inhibition of BK or IK channels would produce an immediate depolarization, reflecting the removal of dominant outward K^+^ conductances active at rest. In contrast, VRAC blockade was predicted to induce hyperpolarization by suppressing a depolarizing Cl^−^ leak (Fig. 8, third row). Reducing extracellular Na^+^ from 140 to 14 mM was predicted to produce biphasic hyperpolarization: an initial rapid component due to loss of Na^+^ leak conductance, followed by a slower hyperpolarizing shift mediated by reduced NKCC-dependent Cl^−^ uptake and progressive intracellular Cl^−^ depletion (Fig. 9 *C*, third row).

**Figure 10:**
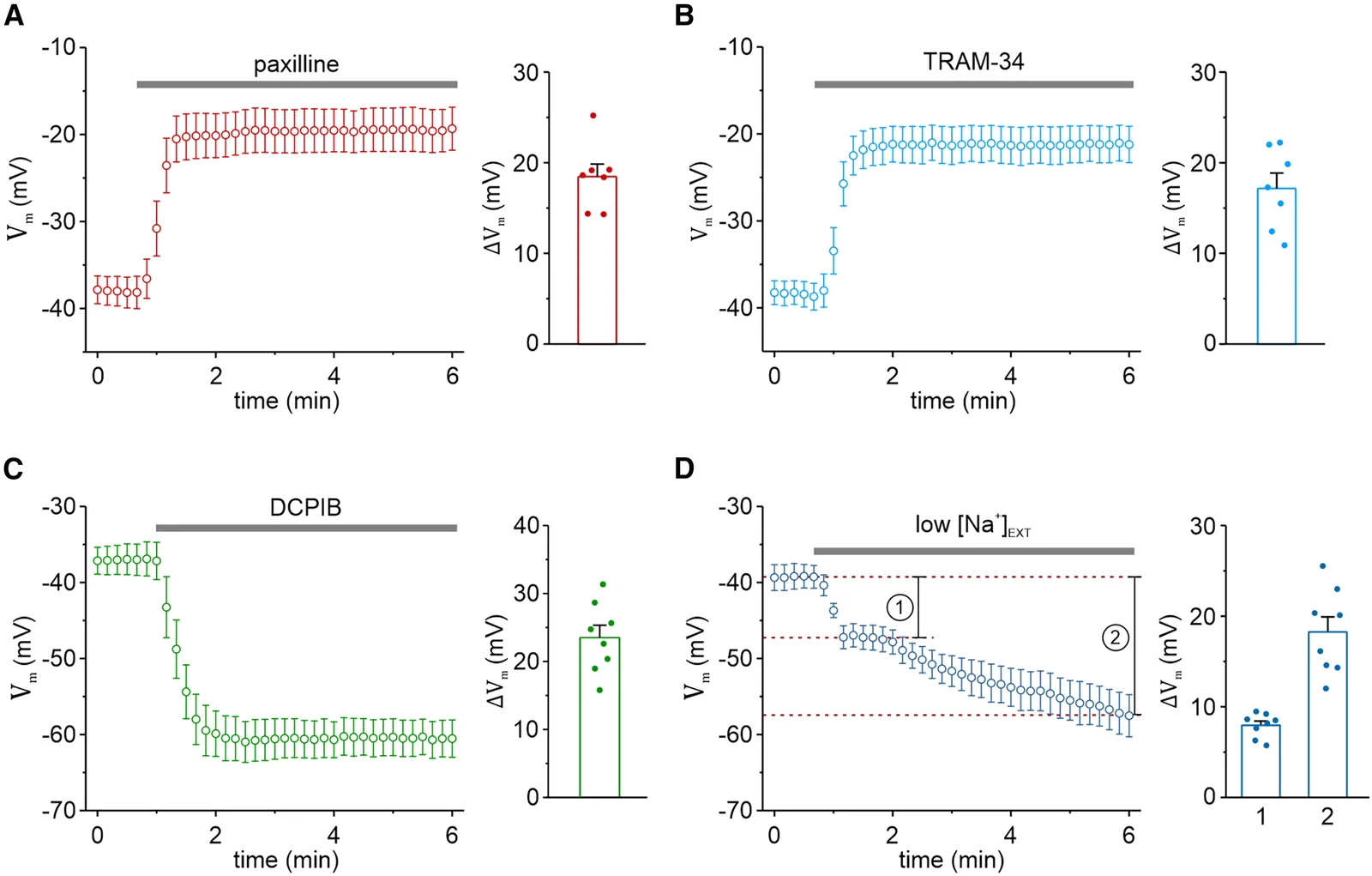
Effects of selective ion channel and transporter blockade on the resting ***V***_***m***_ of U87-MG cells. (*A*–*D*) Average time courses of *V*_*m*_ in U87-MG cells exposed to standard Ringer’s solution supplemented with 1 μM paxilline (*A, red circles*), 3 μM TRAM-34 (*B, cyan circles*), or 10 μM DCPIB (*C, green circles*), selective blockers of BK, IK, and VRAC channels, respectively, or to an isosmotic low-Na^+^ solution containing 14 mM Na^+^ (*D, dark blue circles*). For each condition, the adjacent bar plot reports the mean change in membrane potential (Δ*V*_*m*_) relative to baseline. Pharmacological inhibition of BK (*n* = 7 cells) and IK (*n* = 7 cells) channels produced a rapid, monophasic depolarization, consistent with the loss of major outward K^+^ conductances that contribute to the resting potential. In contrast, VRAC blockade (*n* = 8 cells) elicited an immediate monophasic hyperpolarization, in agreement with the P-L model prediction that VRAC provides a depolarizing Cl^−^ conductance under resting conditions. Reduction of extracellular Na^+^ (*n* = 8 cells) induced a characteristic biphasic hyperpolarization, consisting of an initial rapid shift followed by a slower secondary component. Data are shown as mean ± SEM. All experiments were replicated in at least three independent cultures.

Whole-cell current-clamp recordings qualitatively confirmed the predicted responses. Blockade of BK and IK channels with 1 μM paxilline and 3 μM TRAM-34, respectively, produced rapid and sustained monophasic depolarizations (Fig. 10, *A* and *B*), with mean amplitudes of 18.5 ± 1.4 mV (BK) and 17.2 ± 1.7 mV (IK), indicating that both channels contribute substantially to resting K^+^ conductance. Conversely, VRAC inhibition with 10 μM DCPIB induced a prompt monophasic hyperpolarization of 23.5 ± 1.8 mV (Fig. 10 *C*), consistent with VRAC functioning at rest as a depolarizing Cl^−^ pathway.

Reduction of extracellular Na^+^ to 14 mM via isosmotic substitution with choline^+^ reproduced the biphasic hyperpolarization predicted by the model (Fig. 10 *D*). An initial hyperpolarization of 7.9 ± 0.5 mV occurred within seconds, corresponding to acute suppression of Na^+^ leak, followed by a slower secondary component yielding a total shift of 18.3 ± 1.7 mV. These changes are consistent with model predictions that reduced Na^+^ entry suppresses NKCC activity, lowers intracellular Cl^−^, and drives *E*_*Cl*_ to more negative potentials.

Although the experimental responses matched the qualitative features of the simulations, including depolarization upon BK/IK blockade, hyperpolarization with VRAC inhibition, and biphasic hyperpolarization under low-Na^+^ conditions, quantitative discrepancies were observed. The voltage shifts observed with paxilline, TRAM-34, and DCPIB were slightly larger than model predictions. Additionally, the secondary hyperpolarizing phase under low-Na^+^ conditions developed more rapidly than simulated, indicating that NKCC inhibition and Cl^−^ depletion may occur faster in the experimental system than captured by the simplified P-L model. These differences likely reflect inherent model simplifications, as channel modulation, transporter regulation, or other secondary processes influencing ion homeostasis are not included.

Despite these quantitative deviations, the direction, temporal sequence, and mechanistic origin of all *V*_*m*_ responses were accurately recapitulated by the model. This qualitative concordance strongly supports the robustness of the LP-based parameter estimation and confirms that the P-L model captures the dominant ionic determinants of resting *V*_*m*_ in U87-MG cells.

## Discussion

As emphasized in a recent review by Kay and Blaustein on the origins of the P-L model (1,2), most models of cell excitability focus primarily on ion currents and membrane potential while largely overlooking water fluxes and the resulting dynamic changes in cell volume. This omission is nontrivial, as electrical excitability, ionic gradients, osmotic balance, and cell volume are tightly interdependent. A comprehensive understanding of cellular homeostasis therefore requires explicit integration of electrical and osmotic processes. However, such integration introduces additional dynamical variables described by differential equations governing water movement and intracellular ion concentration kinetics, thereby increasing both mathematical and computational complexity. Faithful modeling of excitable and nonexcitable cells must capture not only transient electrical behavior but also the steady-state and dynamic mechanisms regulating ion fluxes and cell volume . Comparable modeling frameworks have been developed by Vereninov and collaborators, who quantitatively analyzed ionic and osmotic homeostasis in animal cells using computational approaches grounded in charge balance and osmotic equilibrium constraints (40,41).

A major obstacle to the broader adoption of integrated electrical-osmotic models is the challenge of estimating parameters that cannot be directly measured. Classical electrophysiological models often rely on parameters directly measurable using established techniques, whereas many transport processes essential for volume regulation, particularly electroneutral fluxes, cannot be directly quantified with conventional electrophysiology. Realistic model construction therefore requires integrating diverse experimental data through computational inference. Omitting osmotic components can yield physiologically incomplete descriptions, particularly in systems where ionic and osmotic homeostasis are perturbed. Consequently, even simplified models must carefully account for these interdependent processes to generate reliable predictions across varied conditions.

To address this challenge, we developed a method combining steady-state analysis with LP techniques to estimate unknown P-L model parameters from experimentally accessible information. At steady-state, the governing equations reduce to linear relationships among unknown parameters, enabling efficient and constrained estimation. When experimentally measured parameters, such as passive ion conductances, are available, remaining unknown quantities, including transporter and pump fluxes, can be constrained or uniquely determined via LP optimization. We demonstrate the utility of this approach using a simplified P-L model tailored to U87-MG GBM cells. Although the model includes only the major permeant ions (Na^+^, K^+^, and Cl^−^) and a minimal set of transport pathways, it accurately reproduces key experimental observations, including cell swelling following inhibition of specific channels and cell shrinkage under reduced extracellular Na^+^.

The simplified P-L model was chosen to prioritize methodological accessibility and reproducibility of the proposed approach. Nevertheless, the LP-based framework is readily extensible to more physiologically detailed models incorporating additional ions, transport pathways, and kinetic schemes. For example, explicit inclusion of Ca^2+^ dynamics would be particularly relevant for U87-MG cells (11,12) and for systems in which Ca^2+^-dependent transporters contribute to volume regulation. Likewise, more elaborate kinetic schemes, such as multisite binding models or electrogenic transporters, can be incorporated to improve predictions of transient responses. Although such extensions may violate linearity and thus limit direct application of LP , they substantially increase physiological realism. For instance, whereas more complex representations of the Na^+^/K^+^-ATPase have minimal impact on steady-state behavior, they can significantly alter predictions of dynamic responses and regulatory kinetics (13). Similarly, the present model does not incorporate the Ca^2+^ dependence of IK and BK channels, nor the voltage dependence of BK channels. All transmembrane fluxes are treated as voltage independent and steady state, and the intrinsic voltage sensitivity of the Na^+^/K^+^-ATPase is omitted. These simplifications do not substantially affect steady-state predictions under near-physiological conditions but become limiting during rapid transients or strong depolarizations. In vivo, Na^+^/K^+^-ATPase activity is further modulated by β-subunits and FXYD proteins, which tune ion affinity, turnover rate, and voltage dependence (34,42). Incorporating such regulatory mechanisms would likely improve predictive accuracy across a broader range of physiological and pathological contexts, while preserving the core inverse-estimation strategy introduced here.

A key limitation of the proposed LP-based method is that it applies only to parameters that enter linearly into the steady-state equations. Nonlinear parameters, such as the intracellular Na^+^ binding constant of the Na^+^/K^+^-ATPase (*k*_*p*_), must be determined independently and treated as fixed parameters during LP analysis. Similarly, parameters that do not affect the steady-state solution remain unconstrained (undetermined). In our model, this limitation is exemplified by the *P*_*w*_, which governs the rate of volume change but do not influence the final steady-state values of intracellular ion concentrations, membrane potential, or cell volume.

Despite these limitations, nonlinear or fixed parameters tipically correspond to intrinsic kinetic constants of specific transporters or pumps. Such parameters are often conserved across cell types and can be determined experimentally or obtained from the literature, including measurements from heterologous expression systems. In contrast, cell-type-specific quantities, such as ion channel conductances or transporter densities, enter linearly into the steady-state equations of the P-L model and are therefore well suited to LP-based estimation. This feature makes the method particularly powerful for quantifying parameters that are otherwise difficult or impossible to measure experimentally, enabling data-driven, cell-specific characterization of ionic and volumetric homeostasis.

We applied this LP framework to a simplified P-L model of U87-MG cells, a system we have been investigating with respect to the relationship between ion conductances and cell volume regulation (11,12). Using electrophysiological and imaging approaches, we determined several key steady-state variables, including resting *V*_*m*_, baseline cell volume, electrogenic pump activity, and membrane water permeability. These measurements enabled estimation of electroneutral flux parameters through LP optimization. Dynamic simulations of perturbations in ion conductances and extracellular Na^+^ reproduced the direction of experimentally observed cell volume changes and closely matched their time courses, although quantitative discrepancies in magnitude and kinetics were observed in some conditions. These differences likely reflect simplifications inherent in the model, including omission of VRAC volume sensitivity, Ca^2+^ and/or voltage dependence of IK and BK channels, and intracellular Ca^2+^ dynamics. A more comprehensive model incorporating these elements is currently under development in our laboratory.

Interestingly, despite its structural simplicity, the P-L model captures both steady-state and dynamic behaviors of U87-MG cells with remarkable accuracy. This suggests that, under near-physiological conditions, ion and water homeostasis is dominated by the interplay between passive conductances and the electrogenic Na^+^/K^+^-ATPase. More complex regulatory processes, such as signaling-dependent channel modulation, intracellular ion buffering, or metabolic feedback, appear to play a secondary role under basal conditions, becoming functionally relevant primarily during stress or adaptive cellular responses. This minimal P-L framework thus highlights the robustness of pump-leak balance as a core mechanism of cellular homeostasis.

Dynamic regulation of cell volume has important implications for human disease. Pathological conditions including cancer, ischemia, and edema are characterized by rapid or sustained volume changes driven by osmotic stress, ionic dysregulation, or transporter dysfunction (43,44,45). GBM cells, such as U87-MG, actively regulate volume and morphology to migrate through brain tissue (11,12,46), a process requiring coordinated regulation of ion transport and cellular mechanical compliance, as demonstrated by Venkova and colleagues (47). Similarly, ischemic or excitotoxic neuronal injury involves rapid ionic and osmotic shifts that trigger cytotoxic cell swelling. Understanding and predicting these disease-relevant volume changes therefore require integrative models that capture the coupled dynamics of ion transport, osmotic gradients, and water flux.

The LP-based approach presented here provides a practical and generalizable method for estimating key parameters governing ion and volume homeostasis, even when many transport processes are experimentally inaccessible. By enabling systematic, data-driven estimation of steady-state parameters, it supports predictive modeling of disease-related volume dynamics, facilitates hypothesis generation, and informs the design of strategies targeting ion transport. In cancer biology, such models can be used to evaluate how modulation of specific pathways impacts cell migration and osmotic responsiveness. In neurobiology, they can help predict cellular swelling during ischemia or excitotoxic stress.

In summary, we introduced a novel LP-based framework for estimating key parameters of the P-L model from experimentally accessible steady-state data. This approach addresses a central limitation in modeling coupled ionic and osmotic regulation by enabling systematic, data-driven parameter identification without introducing excessive computational complexity. Although demonstrated here using a simplified P-L model of U87-MG cells, the method is readily generalizable to more physiologically detailed systems. By integrating electrical, ionic, and osmotic dynamics, this framework improves both the relevance and predictive power of computational models of cellular homeostasis.

## Data and code availability

The software implementing the LP method proposed in this study, together with the C code used to generate it, is available as supporting material at the GitHub repository: https://github.com/luigicatacuzzeno/LP_for_PL/. Additional data that support the findings of this study are available from the corresponding authors upon reasonable request.

## Acknowledgments

This work was supported by the Italian Ministry of University and Research (MUR) under the PRIN 2022 program (award number: 20223XZ5ER), entitled *“*Kinetic models of ion channels: from atomic structures to membrane currents.” The authors thank Dr. Maria Vittoria Leonardi, Dr. Angela Di Battista, and Prof. Gabriele Di Sante (University of Perugia) for their valuable technical assistance and Prof. Fabio Franciolini for his careful reading of the manuscript and insightful discussions.

## Author contributions

L.C. and A.M. conceived and designed the study and co-wrote the original draft of the manuscript. L.C. performed all simulations and developed the LP framework for parameter estimation. A.M. conducted experiments to determine steady-state variables and parameter constraints, carried out experimental validations for model comparison, and analyzed the data. M.G.C. contributed to the design and interpretation of simulations, discussed the results with L.C. and A.M., and critically revised the manuscript for intellectual content and clarity. All authors reviewed and approved the final version of the manuscript.

## Supporting material

Data S1. The software implementing the LP method proposed in this study, including the executable code and the associated C source code used to generate it The material allows reproduction of the numerical analyses and simulations presented in the manuscript. Detailed instructions for compilation and use are provided within the repository.

Document S2. Article plus supporting material
