# Supplementary material for: An approach based on linear programming to build experimentally driven pump-leak models
Source: Biophys J. 2026 Jan 13;125(4):1165–84. doi: 10.1016/j.bpj.2026.01.021 (PMC13351560; doi:10.1016/j.bpj.2026.01.021)
Supplement: Data S1. The software implementing the LP method proposed in this study, including the executable code and the associated C source code used to generate it — The material allows reproduction of the numerical analyses and simulations presented in the manuscript. Detailed instructions for compilation and use are provided within the repository. [file mmc1.pdf]

Biophysical Journal, Volume *125*

Supplemental information

An approach based on linear programming to build experimentally driven pump-leak models

Luigi Catacuzzeno, Maurizio G. Cavaliere, and Antonio Michelucci

SUPPLEMENTARY DATA. A software implementing the LP method proposed in this study, together with the C code used to generate it, can be found as Supplementary Material at the GitHub repository (https://github.com/luigicatacuzzeno/LP_for_PL/).
